# V_2_CT_x_ MXene as a Sacrificial Promoter for NiFe Catalyst for Anion Exchange Membrane Electrolyzers

**DOI:** 10.1002/advs.75676

**Published:** 2026-05-18

**Authors:** Bastian Schmiedecke, Can Kaplan, Karuppasamy Dharmaraj, Axel Zuber, Merve Buldu‐Aktürk, Ningjun Chen, Mailis Lounasvuori, Xuyun Guo, Valeria Nicolosi, Thorsten Schultz, Norbert Koch, Johanna Rosen, Michelle P. Browne

**Affiliations:** ^1^ Helmholtz Young Investigator Group Electrocatalysis: Synthesis to Devices Helmholtz‐Zentrum Berlin für Materialien Und Energie GmbH Berlin Germany; ^2^ Materials Design Division, Department of Physics, Chemistry and Biology (IFM) Linköping University Linköping Sweden; ^3^ Helmholtz Young Investigator Group Nanoscale Solid−Liquid Interfaces Helmholtz‐Zentrum Berlin für Materialien Und Energie GmbH Berlin Germany; ^4^ School of Chemistry, CRANN and AMBER Research Centers Trinity College Dublin Dublin 2 Ireland; ^5^ Helmholtz‐Zentrum Berlin Für Materialien und Energie GmbH Berlin Germany; ^6^ Institut Für Physik & CSMB Humboldt‐Universität Zu Berlin Berlin Germany

**Keywords:** charge transfer, electronic structure, operando spectroscopy, oxygen evolution reaction, V_2_CT_x_ MXene, water electrolysis

## Abstract

Nickel‐iron layered double hydroxides (NiFe‐LDH) show excellent activity, their poor conductivity limits practical implementation in electrolysers. Previous reports have shown that combining nickel‐iron layered double hydroxide (NiFe‐LDH) materials with MXenes significantly increases the oxygen evolution reaction (OER) activity, however, the rationale behind this is not clear. Herein, we report operando X‐ray absorption spectroscopy (XAS) of V_2_CT_x_ MXene‐enhanced NiFe‐LDH catalysts, revealing important insights into MXene‐hydroxide synergy. Operando X‐ray absorption reveals a two‐step vanadium transformation: V_2_CT_x_ initially oxidizes during hydrothermal synthesis, acting as a reducing agent that promotes ordered Fe_2_NiO_4_ formation, then undergoes further oxidation under OER conditions, to form mixed‐valence V^3+^/^4+^/^5+^ oxide species that establish transient electronic coupling with NiFe active sites. Finally, the NiFe@V_2_CT_x_ composites were tested in an Anion Exchange Membrane (AEM) electrolyzer over 144 h of continuous operation at 500 and 1000 mA cm^−2^, with NF25 achieving degradation rates as low as 0.76 mV h^−1^ at 1000 mA cm^−2^. Postmortem tests reveal that V_2_CT_x_ undergoes progressive dissolution during operation, yet the structurally modified NiFe electrodes retain superior activity relative to the unmodified reference throughout the full test duration. These findings demonstrate that V_2_CT_x_ functions beyond passive conductive support as an active electronic participant whose structural legacy sustains durable performance even after vanadium leaching.

## Graphical abstract

These findings demonstrate that V_2_CT_x_ functions beyond passive conductive support as an active electronic participant whose structural legacy sustains durable performance even after vanadium leaching in Anion Exchange Membrane (AEM) Electrolysers.

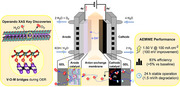

## 1 Introduction

The global transition toward sustainable energy systems has positioned green hydrogen production via water electrolysis as a cornerstone technology for achieving carbon neutrality by 2050 [1, 2]. While anion exchange membrane water electrolysis (AEMWE) offers the advantage of using non‐precious metal catalysts compared to proton exchange membrane (PEM) systems, current AEMWE technologies struggle to achieve low degradation rates during operation which limits commercial scalability. In order to achieve improved stability, the Strategic Research and Innovation Agenda (SRIA) of the Clean Hydrogen Partnership has set a 2030 target of 0.5% over 1000 h for AEM electrolyzers [3].

The oxygen evolution reaction (OER) at the anode is the primary efficiency bottleneck involving multiple electron transfers. Among the various transition metal‐based catalysts, nickel‐iron layered double hydroxides (NiFe‐LDHs) are emerging as the most promising non‐precious electrocatalysts for AEMWE. During operation, these materials undergo critical structural transformations, converting from the semiconducting α‐Ni(OH)_2_ phase to the conductive, catalytically active γ‐NiOOH phase characterized by Ni^3+^/^4+^ species and Fe^3+^ Lewis acidic sites [4, 5, 6, 7]. Iron plays a crucial role during electrochemical operation in promoting nickel oxidation and stabilizing OER intermediates [4, 5, 6, 7]. While NiFe‐LDHs can achieve competitive overpotentials of 200–250 mV at 10 mA cm^2^ in three‐electrode configuration, their poor electronic conductivity severely limits performance in practical membrane‐electrode assemblies (MEAs) at high current densities over time, creating substantial ohmic losses that prevent reaching commercial targets [8, 9]. The insulating nature of hydroxide structures hinders efficient electron transport during the electrocatalytic process, necessitating strategies to enhance conductivity while maintaining the beneficial NiFe synergy [10].

Traditional approaches including heteroatom doping and carbon supports show promise but often compromise stability [11]. MXenes—two‐dimensional transition metal carbides and nitrides—offer an alternative through metallic conductivity, hydrophilic surfaces, and tunable surface chemistry [12, 13]. Ti_3_C_2_T_x_ MXene has been studied in combination with NiFe systems for oxygen evolution reaction (OER) applications, achieving overpotentials of 270–319 mV, demonstrating the potential of MXene‐supported architectures [14, 15]. Recent evidence suggests that V_2_CT_x_ MXene may offer superior electrochemical properties compared to Ti_3_C_2_T_x_ [16]. V_2_CT_x_ exhibits several key advantages: (i) multiple vanadium oxidation states enabling versatile redox chemistry, (ii) lighter atomic mass and fewer atomic layers per sheet facilitating faster ion diffusion, (iii) larger work function differences that can optimize electronic interactions with metal hydroxides [17], and (iv) when V_2_CT_x_ oxidizes, it can form OER active materials [18], while TiO_2_ is detrimental for the catalytic performance. Previous studies have demonstrated that V_2_CT_x_ incorporation improves NiFe OER activity, with hydrothermally synthesized composites achieving overpotentials of 222–250 mV [19, 20]. However, catalyst durability under practical operating conditions remains inadequately characterized: existing stability assessments are limited to low current densities (≤10 mA cm^−2^) and short durations (≤10 h) in three‐electrode configurations [19, 20], providing insufficient insight into long‐term performance degradation at industrially relevant current densities (>100 mA cm^−2^). Furthermore, the structural evolution of V_2_CT_x_ under oxidizing OER conditions and its dynamic electronic coupling with NiFe active sites remain mechanistically unexplored, hindering rational design of durable, high‐performance water electrolyzers. Despite these promising results, fundamental understanding of V_2_CT_x_‐NiFe catalysts behavior under sustained operation remains incomplete. Three critical questions must be addressed: (i) What is the chemical state and structural evolution of V_2_CT_x_ under OER conditions? (ii) How does V_2_CT_x_ electronically interact with NiFe active sites during OER? (iii) Do the performance improvements observed in 3‐electrode testing translate to practical AEM electrolyzer operation at high current densities? Addressing these questions requires operando spectroscopic investigation coupled with device‐level validation, both absent from prior work [19, 20].

Here, we employ operando X‐ray absorption spectroscopy (XAS) to elucidate the mechanisms underlying the enhancement of NiFe‐LDH@V_2_CT_x_ catalysts under OER conditions, synthesized via a urea‐assisted hydrothermal method. Our investigation reveals that V_2_CT_x_ first oxidizes during hydrothermal synthesis which acts as a reducing agent that promotes ordered Fe_2_NiO_4_ formation, then undergoes further oxidation under OER conditions to mixed valence V^3+^/V^4+^/V^5+^ species, forming dynamic V‐O‐M bridges that electronically couple with NiFe sites. This coupling suppresses detrimental Ni over‐oxidation while maintaining Fe^3+^ coordination. Furthermore, we validate catalyst durability through complementary stability testing: (i) 12 h chronopotentiometry at 100 mA cm^−2^ in 3_‐_electrode configuration, and (ii) 24‐h zero‐gap AEM electrolyzer operation at 100 mA cm^−2^ at 60°C, where V_2_CT_x_‐enhanced NiFe catalysts operated at cell voltages 80 mV lower than pure NiFe over 24 at 100 mA cm^−2^, with NF25 exhibiting a voltage degradation rate 50% lower (1.5 vs. 3.1 mV h^−1^) than that of the pure NiFe catalyst. Extended 144 h testing at 500 and 1000 mA cm^−2^ reveals that degradation rates decline sharply after the initial vanadium‐loss phase, reaching as low as 0.76 mV h^−1^ (NF25) at 1000 mA cm^−2^. Post‐mortem ICP‐OES, EDX, and XPS confirm that V_2_CT_x_ functions as a sacrificial electronic modifier whose lasting effect on the NiFe phase sustains the activity advantage even after complete surface vanadium depletion.

The insights gained in this work establish new design principles for electronically coupled catalyst‐support systems capable of meeting commercial AEMWE performance targets.

## 2 Materials and Methods

### 2.1 Materials

Graphite rod (redox.me), mercury–mercury oxide (Hg/HgO) reference electrode (redox.me), glassy carbon disc electrode (ALS instruments), and Nafion (Sigma Aldrich) were used for electrochemical measurements. Sodium hydroxide pellets (≥98%, reagent‐grade), ethanol (≥99%, reagent‐grade), isopropanol (≥99%, reagent‐grade), Ni(SO_4_)_2_·6 H_2_O (≥99% metal basis, M  =  290.79 g mol^−^^1^), Fe(NO_3_)_3_·9 H_2_O (≥99% metal basis, M  =  404.00 g mol^−1^), tetrabutylammonium hydroxide (TBAOH, 40% in H_2_O), and urea (≥99%, ACS reagent, M  =  60.06 g mol^−1^) were purchased from Sigma‐Aldrich. NaF and HCl were purchased from Lachner. For sample preparation and dilutions, ultrapure water with a resistivity of 18.2 MΩ cm was used.

### 2.2 Material Synthesis

#### 2.2.1 Synthesis of V_2_AlC MAX Phase

The V_2_AlC MAX phase precursor was synthesized from stoichiometric amounts of powdered graphite (99.999%, Sigma Aldrich), vanadium metal powder (99.99%, Sigma Aldrich), and aluminum powder (99.8%, Alfa Aesar). The powders were thoroughly mixed using an agate mortar and pestle to ensure homogeneous distribution. The powder mixture was then transferred to an alumina crucible and heated to 1550°C for 2 h under flowing argon atmosphere to prevent oxidation. Following the high‐temperature treatment, the sample was allowed to cool to room temperature within the furnace, resulting in a loosely packed powder. The obtained powder was subsequently crushed and sieved through a 450‐mesh sieve to achieve uniform particle sizes of approximately 32 µm.

#### 2.2.2 Preparation of Delaminated V_2_CT_x_ MXene

The V_2_CT_x_ MXene was prepared through selective etching of the aluminum layer from the synthesized V_2_AlC MAX phase using hydrofluoric acid. V_2_AlC powder (2 g, 450 mesh) was gradually added to a 48% HF solution (20 mL) under continuous stirring to ensure complete wetting and uniform etching. The mixture was maintained at 40°C under constant stirring for 72 h to achieve complete aluminum removal. The resulting dispersion was centrifuged at 6000 rpm for 5 min to collect the etched material, which was then repeatedly washed with deionized water through centrifugation cycles until the pH of the supernatant reached approximately 6.

For delamination of the multilayered MXene structure, TBAOH was added to facilitate intercalation between the MXene layers. The mixture was manually shaken for approximately 5 min to promote intercalation, followed by centrifugation at 6000 rpm for 5 min. The intercalated material was thoroughly rinsed with deionized water three times without agitation to remove residual TBAOH. The intercalated MXene powder was then dispersed in deionized water (30 mL), manually shaken for 10 min, and centrifuged at 3000 rpm for 30 min. The high‐quality delaminated V_2_CT_x_ MXene suspension was carefully decanted, excluding any remaining sediment, to obtain the final MXene dispersion with a concentration of approximately 1.47 mg mL^−1^ for composite synthesis.

#### 2.2.3 Synthesis of NiFe@V_2_CT_x_

NiFe@V_2_CT_x_ composites were synthesized through a urea‐assisted hydrothermal method. In a typical synthesis, predetermined volumes of V_2_CT_x_ colloidal solution (1.47 mg mL^−1^) were added to a reaction vessel to achieve target weight percentages of 25 and 50 wt.% based on theoretical yields. Specifically, 5.1 mL (7.5 mg) and 10.2 mL (15 mg) of V_2_CT_x_ dispersion were used, respectively. Subsequently, 5 mmol urea, 15 mmol Ni(SO_4_)_2_·6 H_2_O and 5 mmol Fe(NO_3_)_3_·9 H_2_O were added to the solution (maintaining a Ni:Fe ratio of 3:1). The total solution volume was adjusted to 25 mL with deionized water, and the mixture was stirred for 30 min to fully dissolve all the compounds. The solution was then transferred into a Teflon‐lined stainless‐steel autoclave and heated to 120°C for 6 h for hydrothermal treatment. After cooling down to room temperature, the precipitate was collected by centrifugation at 5000 rpm for 10 min, and then repeatedly washed with deionized water (3×) and ethanol (3×). The sediment was dried at 60°C for 10 h. The collected powder samples were labeled as NF25 and NF50, corresponding to target V_2_CT_x_ contents of 25 and 50 wt.%, respectively. The pure NiFe material, labeled NiFe, was synthesized using the same method without addition of V_2_CT_x_.

### 2.3 Methods

#### 2.3.1 Structural Characterization

Powder X‐ray diffraction (XRD) analysis was performed at room temperature (RT) using a Bruker D8 Advance diffractometer with Cu Kα radiation (λ  =  1.5406 Å), operated at 40 mA and 40 kV, for the synthesized materials. Diffraction patterns were collected over a 2θ range of 5° to 90°, with a step size of 0.02°. The 5°–70° region is presented in the paper, as no diffraction peaks were observed beyond 70°. Phase identification was performed using the Crystallography Open Database (COD), with the carbonate‐intercalated hydrotalcite structure (Crystallography Open Database, COD ID 2102792: Mg_6_Al_2_(OH)_16_CO_3_·4 H_2_O) serving as a structural template with Mg^2+^/Al^3+^ substituted by Ni^2+^/Fe^3+^ to model the NiFe‐CO_3_‐LDH phase. Morphological analysis was carried out using a Zeiss MERLIN field emission scanning electron microscope (FESEM) with a GEMINI II optical column using an accelerating voltage of 2 kV, equipped with a secondary electron detector. Energy‐dispersive X‐ray spectroscopy (EDX) analyses were conducted using an Oxford Instruments Ultim Extreme EDX detector. Elemental mappings were acquired at an accelerating voltage of 10 kV. EDX peaks were identified using AZtecLive software from Oxford instruments. Transmission electron microscopy (TEM) and high‐angle annular dark field scanning TEM (HAADF‐STEM) were performed using uncorrected FEI Titan with Schottky field emission S‐FEG source operated at 300 kV. Electron energy‐loss spectroscopy (EELS) mapping was carried out with Quantum Gatan Imaging Filter (GIF) detector with energy dispersion of 0.5 eV per channel.

X‐ray photoelectron spectroscopy (XPS) measurements were conducted with a JEOL‐9030 setup, using a non‐monochromatic Mg source for excitation and a pass energy of 20 eV, yielding an overall energy resolution of 1.0 eV. The powders were deposited on carbon tape, and the binding energy scale was referenced to the C1s peak of the carbon tape set to 285.0 eV, as the sample showed charging. Quantitative deconvolution was performed exclusively on the Ni2p_3/2_, Fe2p_3/2_, and V2p core level regions, following established practice for first‐row transition metal XPS analysis [21, 22, 23].

Inductive Coupled Plasma (ICP‐OES) measurements were conducted with an iCAP 7400 DV (ThermoFisher) in axial measurement mode.

FTIR spectra were measured in the attenuated total reflectance (ATR) mode with a Bruker Alpha spectrometer equipped with a room temperature DLaTGS detector and a diamond ATR accessory. The clean diamond crystal was used as the reference. All spectra were accumulated for 128 scans. The resolution was 2 cm^−1^.

#### 2.3.2 Electrochemical Measurements

The electrochemical performance of the materials was characterized in a standard three‐electrode cell configuration in alkaline aqueous solution at room temperature. 1.0 M NaOH electrolyte was prepared from NaOH pellets and degassed with nitrogen for 10 min before measurements. A standard mercury–mercury oxide electrode (Hg/HgO) was employed as the reference electrode (RE) and a graphite rod as the counter electrode (CE). The working electrode was a 3 mm diameter glassy carbon (GC) disk with a geometric surface area of 0.0707 cm^2^. The cell was connected to an electrochemical workstation (CHI760E) and a rotating ring disc electrode (RRDE, ALS‐Japan). The GC electrode was polished using 0.05 µm alumina polish before each use to ensure a clean surface. A catalytic ink was prepared for every material by dispersing 10 mg of catalytic powder in 1.0 mL DI water/isopropanol solution (1:1) and 8 µL Nafion. The solution was ultrasonicated for 10 min to form a homogeneous ink. To reach a catalyst loading of 0.16 mg cm^−2^, 1.13 µL ink was drop‐casted onto the polished GC disk and allowed to dry in air at room temperature. All measurements were performed in nitrogen saturated 1 mol L^−1^ NaOH electrolyte. Cyclic voltammetry (CV) was conducted between the potential ranges of 0.63 V and 1.58 V vs. RHE. Each catalyst was subjected to 5 CV cycles in the potential range at a scan rate of 40 mV s^−1^. Linear Sweep Voltammetry (LSV) and Tafel plot measurements were performed at a scan rate of 1 mV s^−1^ and a rotation rate of 1600 rpm. Polarization data was 100% iR‐drop corrected using the automatic iR compensation (current interrupt method) of the potentiostat. To determine the charge transfer resistance of the materials under operation, EIS measurements were conducted in the OER region at 1.6 V vs. RHE. To fit the impedance data, the modified Randles circuit R_s_(CPE||R_ct_) was used, where CPE represents the constant phase element accounting for the non‐ideal capacitive behavior of the electrode‐electrolyte interface. For electrochemically active surface area (ECSA) determination, electrical double‐layer capacitance (C_dl_) was measured by performing multiple cyclic voltammetry measurements over a 100 mV scan range with scan rates ranging from 1 to 100 mV s^−1^. The respective currents measured in the non‐Faradaic region were plotted against scan rate, and C_dl_ was determined from the slope of this linear relationship. ECSA was calculated by dividing C_dl_ by the specific capacitance (C_s_  =  0.04 mF cm^−2^ for transition metals in 1.0 M NaOH). The mean overpotential at 10 mA cm^−2^ (η_10_) was calculated from at least three independent electrode measurements per material. For long‐term electrocatalytic stability tests, chronopotentiometry was conducted at constant current densities of 100 mA cm^−2^ for 12 h. Nickel fiber felt (1.0 cm^2^, 35 µm fiber diameter, 0.5 mm thickness, 80% porosity, Xinxiang Aida Machinery Equipment Corporation, China) was used as the working electrode substrate. The nickel fiber felts were cleaned by sonicating in 20% HCl for 10 min, followed by sequential sonication in deionized water, acetone, isopropanol, ethanol, and deionized water for 10 min each. The cleaned substrates were then spray‐coated with 1.0 mg of catalytic material before electrochemical testing.

#### 2.3.3 AEM Electrolyzer Tests

Zero‐gap AEM water electrolysis tests were performed using a ZAHNER ZENNIUM PRO potentiostat coupled with a PP211 booster which was controlled by Thales software. A commercial X‐Cell electrolyzer (Redox Flow) with a geometric active area of 6.25 cm^2^ (2.5 cm × 2.5 cm) was employed. The cell was operated at 60°C in 1.0 M NaOH electrolyte, with nickel and stainless‐steel current collectors at the anode and cathode, respectively. Hydrogen production was quantified using a Bronkhorst EL‐FLOW Select mass flow meter/controller (100 mL min^−1^), preceded by a silica gel drying column (Merck Supelco, orange granulate, 1–3 mm) to remove moisture. The electrolyte was recirculated co‐flow at 25 mL min^−1^ to both anode and cathode compartments from each separate reservoirs via a dual‐channel BT600L Intelligent Flow peristaltic pump (Lead Fluid). Cell temperature was maintained at 60°C using a Juchheim LTR 2500 temperature controller. Tygon A‐60‐G tubing (I.D.  =  4.3 mm, O.D.  =  6 mm), and an RS PRO cylindrical heating mat (138 W, max. 200°C, Ø 115 mm, 240 V AC) were used. The electrolyzer setup is shown in Figure S1. Nickel fiber felt (NF, 35 µm fiber diameter, 0.5 mm thickness, 80% porosity, Xinxiang Aida Machinery Equipment Corporation, China) served as the high‐surface‐area metallic gas diffusion layer (GDL) substrate for both electrodes. Catalyst inks were prepared with a 1:3 water : isopropanol ratio (total volume 1.0 mL) and a nafion (5 wt.%) loading of 8 µL mg^−1^_catalyst_, ultrasonicated for 30 min, and spray‐coated onto the 2.5 cm × 2.5 cm area NF GDL using an N_2_‐pressurized gun (1.0 bar). Anode and cathode catalyst loadings were 1.0 mg cm^−2^ and 0.5 mg_Pt_ cm^−2^, respectively. A PiperION A80 of thickness 80 µm anion exchange membrane (AEM) from Versogen was used, pre‐activated by immersion in N_2_‐saturated 1.0 M NaOH for ≥ 24 h. Ethylene propylene diene monomer gaskets (0.5 mm thickness) sealed the anode and cathode compartments, with assembly torque applied in a zigzag pattern, incrementally increasing from 0 to 5.0 Nm in 1.0 Nm steps. Steady‐state polarization curves for *I‐V* curve were obtained via chronopotentiometry, holding each current density for 30 s and averaging the final 5 s. High‐frequency resistance (HFR) measurements, representing ohmic cell resistance, were conducted using galvanostatic electrochemical impedance spectroscopy (GEIS) from 200 to 1 kHz at 100 mA cm^−2^ with a 10% current amplitude (minimum 5 mA cm^−2^). HFR values were extracted from the high‐frequency x‐intercept. Full GEIS spectra were recorded from 200 kHz to 100 mHz.

Electrolyzer efficiency calculations:

The theoretical H_2_ production volume in L is given by

(1) $$V_{H_{2}}=\frac{V_{mol}\;\times \;Q}{z\;\times \;F}\;$$

where *V_mol_*, *Q*, *z*, and *F* represent the molar volume of the gas under standard conditions (22.4 L mol^−1^), total charge passed (Q  =  I × t in C), number of electron transfer (Z  =  2), and Faraday constant which is 96485 C mol^−1^, respectively.

The energy consumption in J L^−1^ is given by

(2) $$W=\frac{U\;\times \;I\;\times \;t}{V_{H_{2}}}\;$$

where *U*, *I*, *t*, and $V_{H_{2}}$ represent the working electrolyzer voltage in V, working total current in A (electrode area in cm^2^ × current density in A cm^−2^), total time of applied cell voltage or current in s, and theoretical H_2_ production volume in L. The density of H_2_ is 0.089 g L^−1^. Here we used the mean electrolyzer voltage from 24 h stability tests.

Conversion factor from J L^−1^ to kWh kg^−1^ is

(3) $$\mathrm{kWh}\;kg^{-1}=JL^{-1}\;\times \;0.0031191$$

The electrolyzer power in W cm^−2^ is given by

(4) $$EP=U\times \frac{I}{electrode\;area\;in\;cm^{2}}$$

H_2_ power in W cm^−2^ is given by

(5) $$HP=H_{2}\;\;production\;rate\times \;LHV$$

where LHV is the low heating value which is 2.42 × 10^5^ J mol^‒1^ for H_2_.

The H_2_ production rate in mol H_2_ cm^−2^ s^−1^ is given by

(6) $$H_{2}\;production\;rate=\frac{current\;density\;in\;A\;cm^{-2}}{2F}\;$$

The Cell efficiency (CE) in % is given by

(7) $$CE=\frac{HP}{EP}\;\times 100\%$$

### 2.4 X‐Ray Absorption Spectroscopy Data Collection

X‐ray Absorption Spectroscopy measurements were conducted under synchrotron radiation at SOLEIL beamline SAMBA (France). After appropriate calibration of the X‐ray beam, the materials were drop‐casted on graphite foil before being installed in a commercial electrochemical cell. Spectra were collected at the Ni K‐edge (8333 eV), Fe K‐edge (7112 eV), and V K‐edge (5465 eV). The resulting fluorescence emission was acquired using a liquid nitrogen‐cooled 2D detector at 30–150 cm distance from the samples in fluorescence mode. The operando measurements were performed using 1.0 M NaOH in a 3‐electrode setup controlled by a Biologic SP‐200 electrochemical workstation, the reference was a commercial Hg/HgO electrode, and the counter electrode was a platinum wire. A schematic drawing of the experimental setup is provided in Figure S2. All samples were characterized before and after the OER conditions (ex situ/OCP), as well as at different potentials between 0 and 0.9 V vs. RHE which could strategically vary according to the absorption edge and sample nature. Spectra for each absorption edge were recorded at least 3 times to ensure adequate signal‐to‐noise ratio, although abundant bubble formation or film degradation under potential could lessen the quality of the data and constrain the results analysis.

### 2.5 X‐Ray Absorption Spectroscopy Data Analysis

The µ(E) data were processed with the DEMETER software suite [24]. The absorption edges pre‐ and post‐edge lines as well as the maximum of the first derivative were first defined in Athena to normalize the edge step and obtain χ(E). The normalized edges were further plotted, and the edge position was arbitrarily defined as the value at the half maximum of the normalized step (0.5) to proceed with the XANES analysis. Artemis was used to plot the normalized data Fourier Transform χ(R) and perform a first shell analysis in the R = [1;2] and k = [2;8+] region defining oxygen (or carbon when relevant) as the first absorber [6, 7, 25]. The fitting results were considered satisfactory according to standard criteria for these tools: R‐factor <0.02, ΔE_0_ typically between ± (0–15) eV, S_0_^2^ in the range of 0.7–1.05, and σ^2^ positive typically in the range of 0.003‐0.015 Å^2^ [26].

## 3 Results and Discussion

### 3.1 Structural Characterization

**FIGURE 1:**
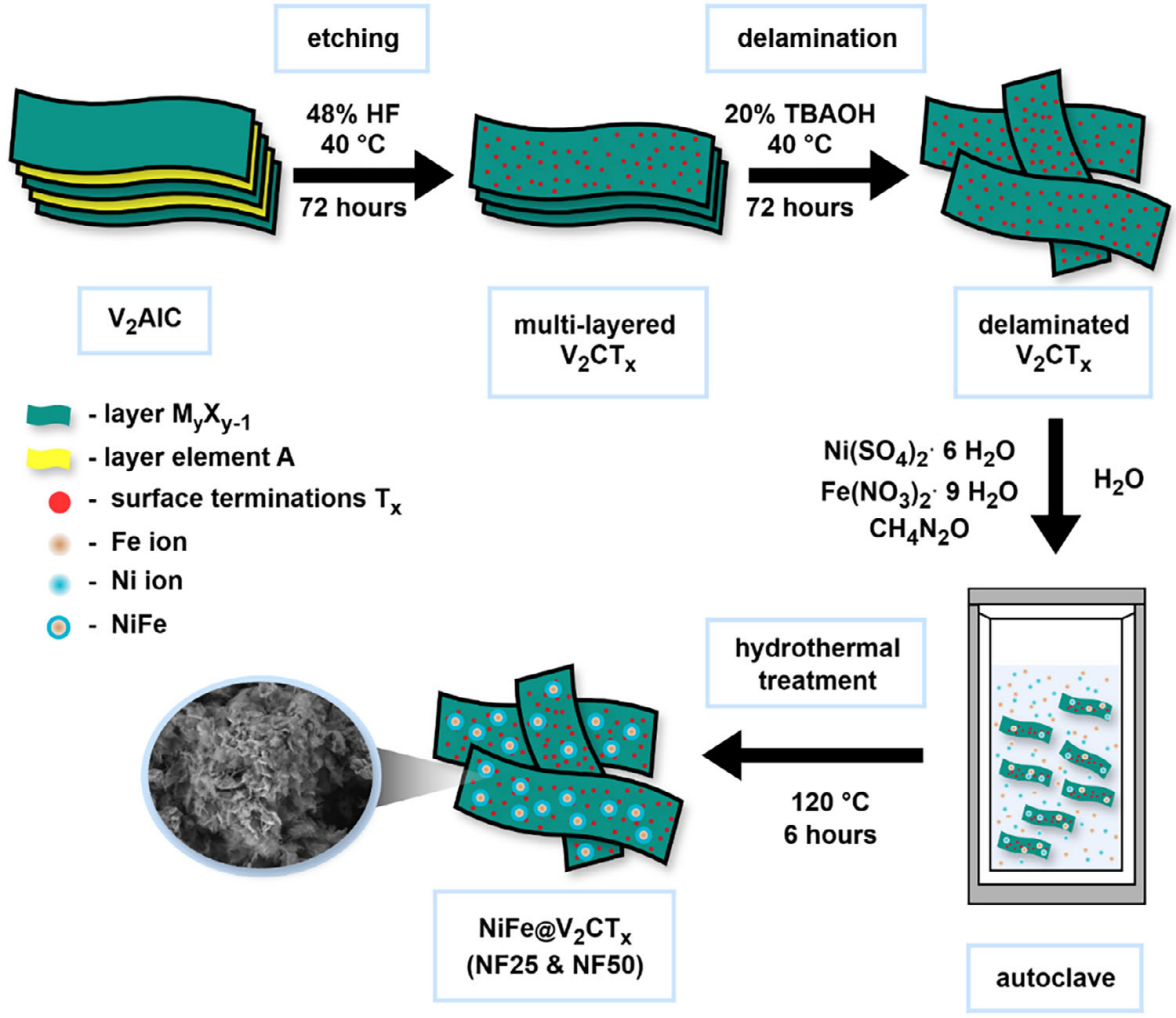
Synthesis schematic for the fabrication of the NiFe@V_2_CT_x_ composites.

The synthesis of NiFe@V_2_CT_x_ composites followed a dual‐step hydrothermal strategy with urea‐assisted precipitation, as depicted in Figure 1. V_2_CT_x_ MXene was initially prepared by selective aluminum extraction from V_2_AlC using 48 wt.% HF, producing accordion‐like multilayered V_2_CT_x_. Delamination in 20% tetrabutylammonium hydroxide (TBAOH) solution yielded dispersed V_2_CT_x_ nanosheets. In the second step, various weight percentages of the delaminated V_2_CT_x_ were subsequently added to an autoclave containing Ni(SO_4_)_2_·6H_2_O, Fe(NO_3_)_3_·9H_2_O, urea, and distilled water. Hydrothermal synthesis was then conducted at 120°C for 6 h, facilitating the nucleation and growth of metal hydroxides on the MXene surface. The concentration of V_2_CT_x_ in the composite materials were 25 and 50 wt.% and the resulting composite catalysts will be denoted as NF25 and NF50 from here on. A pure NiFe catalyst was synthesized under identical reaction conditions just without the addition of V_2_CT_x_. To identify the chemical and structural properties of the prepared catalysts before the OER studies, the materials were characterized by XRD, SEM, EDX, and XPS.

#### 3.1.1 Scanning Electron Microscopy (SEM) and Energy Dispersive X‐Ray Spectroscopy (EDX)

**FIGURE 2:**
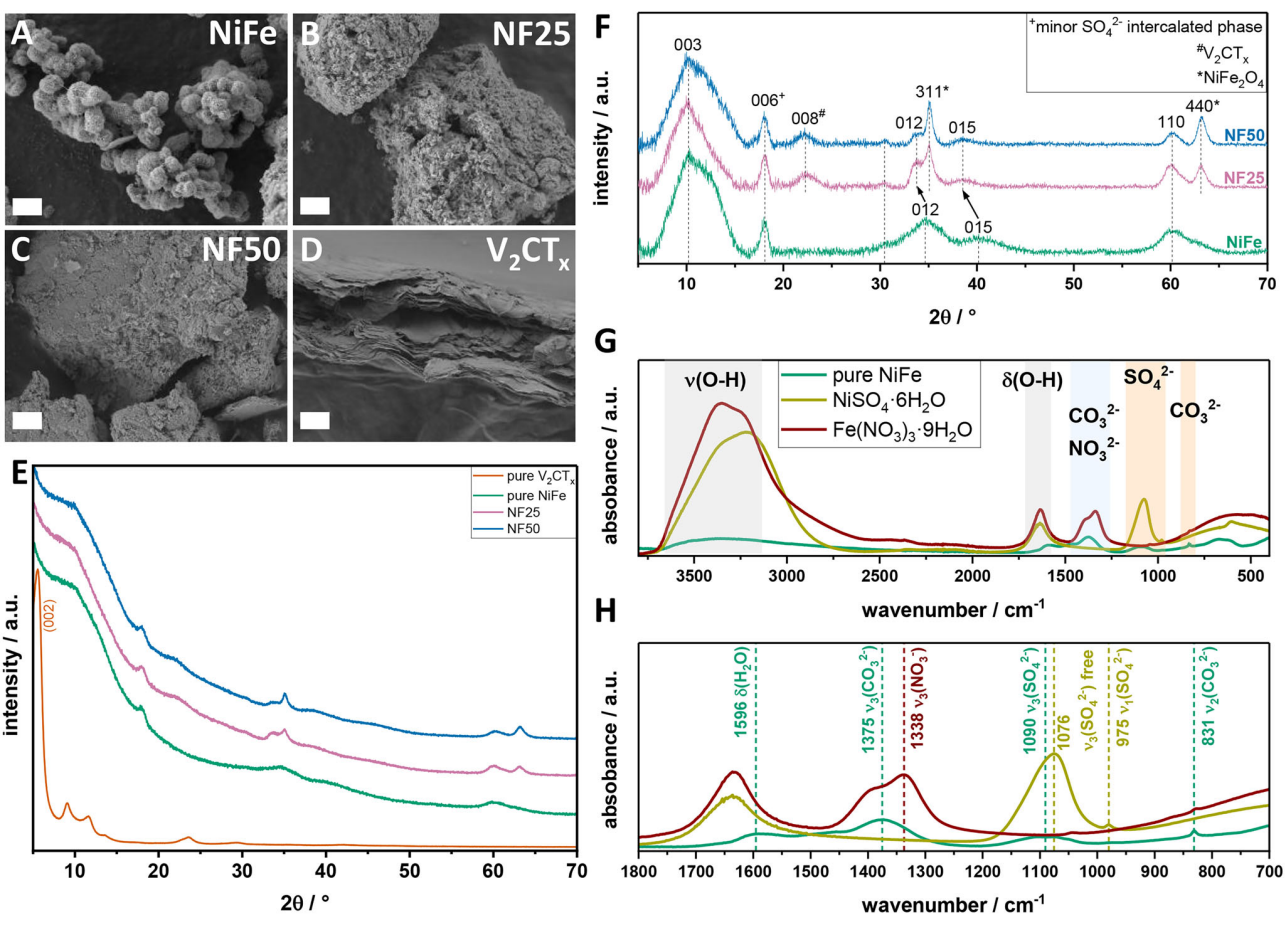
Morphological and structural characterization of NiFe@V_2_CT_x_ composites. Scanning electron microscopy images of (A) pure NiFe showing urchin‐like microstructures, (B,C) NiFe@V_2_CT_x_ composites at 25 wt.% (NF25) and 50 wt.% (NF50) loading, respectively, exhibiting altered morphology, and (D) pure V_2_CT_x_ showing characteristic layered MXene structure. Scale bars: 2 µm. (E) Raw powder X‐ray diffraction patterns of pure NiFe, pure V_2_CT_x_ MXene, and NiFe@V_2_CT_x_ with 25 wt.% (NF25), and 50 wt.% (NF50) V_2_CT_x_ loading. (F) Background‐subtracted XRD diffractograms. (G) FTIR spectra (4000‐250 cm^−1^). (H) Expanded fingerprint region (1800–700 cm^−1^) with peak positions and band assignments.

Scanning Electron Mycoscopy (SEM) was carried out to investigate the morphological and microstructural characteristics of the synthesized materials. Clear morphological differences were observed between the pure and hybrid catalysts. The pure NiFe catalyst presents urchin‐like microstructures, in which numerous rod‐like nanostructures grow outward from the core, typical for various metal oxides and hydroxides [27], Figure 2A. The microstructure was significantly altered upon the introduction of V_2_CT_x_ into NiFe, as shown in Figure 2B,C. The urchin‐like structures are no longer visible in the NiFe@V_2_CT_x_ hybrids. With abundant nucleation sites on the surface, the V_2_CT_x_ sheets facilitated random nucleation and growth of NiFe, and the microstructure became a mixture of aggregated NiFe and 2D V_2_CT_x_ sheets. This change indicates that the incorporation of V_2_CT_x_ alters nucleation and growth dynamics during synthesis. The pure V_2_CT_x_ exhibits multilayered sheets typical of MXene materials, as shown in Figure 2D.

Energy Dispersive X‐ray (EDX) analysis (Figure S3) was performed to investigate the elemental compositions and the distribution of NiFe and V_2_CT_x_ among the composites. EDX elemental mapping confirms that the fluorine‐terminated V_2_CT_x_ was successfully incorporated into the NiFe system, and NiFe was homogeneously decorated onto MXene sheets across hybrid compositions, Figure S3B,C. Additionally, EDX mappings reveal minor oxidation on the V_2_CT_x_ surface, as well as the presence of residual Al in the V_2_CT_x_, in accordance with the XRD results.

#### 3.1.2 X‐Ray Diffraction Analysis

X‐ray diffraction (XRD) analysis was carried out to investigate the phases present in the synthesized pure and hybrid electrocatalysts, and the effects of incorporating V_2_CT_x_ into NiFe at loadings of 25 and 50 wt.%, shown in Figure 2E. All d‐spacings were calculated using Bragg's law. Peak positions were assigned from Diffrac.EVA background‐subtracted diffractograms (Bruker; Figure S4A) and all reported 2θ values and derived d‐spacings correspond to the corrected patterns. Raw diffractograms are shown in Figure 2E for reference.

The pure NiFe material exhibits characteristic reflections of carbonate (CO_3_^2^^−^)‐intercalated layered double‐hydroxide (LDH) (Crystallography Open Database, COD ID 2102792: Mg_6_Al_2_(OH)_16_CO_3_·4 H_2_O). The dominant broad peak at 10.28° 2θ corresponds to the (003) basal reflection with d‐spacing of 8.60 Å, consistent with hydrothermally grown carbonate‐intercalated LDH structures. This interlayer distance falls between literature values for dry CO_3_^2^^−^‐LDH (d_003_  =  7.56–7.85 Å) and NO_3_‐LDH (d_003_  =  8.79–9.02 Å), consistent with a partially hydrated interlayer in which co‐intercalated water molecules expand beyond the dry carbonate value, as commonly observed in hydrothermally synthesized LDHs [28, 29] From the (003) and (110) reflections, lattice parameters of *a* = 3.077 Å and *c* = 25.79 Å were derived, in good agreement with reported values for Ni^2^^+^/Fe^3^^+^ LDHs [30, 31] Subtracting the brucite‐like layer thickness of 4.77 Å from d_003_ yields an interlayer gallery height of 3.83 Å, consistent with hydrated carbonate intercalation. Additional LDH reflections at 34.55° (012), 40.46° (015), and 60.10° (110) confirm the phase assignment; the (110) reflection is particularly diagnostic as it directly reports the in‐plane metal–metal distance and is insensitive to interlayer composition. A minor reflection at 18.04° does not correspond to the (006) harmonic of the main CO_3_^2^^−^‐LDH phase, which would be expected at 21.0°, and is instead attributed to the (006) reflection of a co‐intercalated minor SO_4_^−2^‐LDH phase (implied d_003_  =  9.83 Å, at the upper boundary of the reported range of 8.71–9.80 Å for sulfate‐intercalated LDH) [30, 31], attributable to partial retention of sulfate from the NiSO_4_ precursor alongside the dominant carbonate anion. This assignment is independently corroborated by two FTIR signatures: the ν_3_(SO_4_^2^^−^) band at 1100.7 cm^−1^ (blueshifted +25.5 cm^−1^ from free NiSO_4_·6H_2_O, confirming gallery intercalation) and the symmetry‐activated ν_1_(SO_4_^2^^−^) band at 975 cm^−1^, which is IR‐inactive in free tetrahedral sulfate but becomes allowed upon reduction to C_2v_/C_s_ symmetry upon intercalation (Figure 2G,H) [32, 33]. A weak, broad reflection at ∼30.4°, present as a shoulder in all samples, could not be unambiguously assigned and is consistent with either a higher‐order LDH stacking harmonic or a minor spinel (220) contribution (see Table S1); it is therefore left unassigned. The overall breadth of the LDH reflections is consistent with turbostratic stacking disorder and nanoscale crystallite size, both characteristic of hydrothermally synthesized LDHs [28, 29].

In the case of pure V_2_CT_x_, Figure 2E, a strong peak is observed at a low 2θ angle of 5.5°, confirming the MXenes characteristic (002) basal reflection. Additional significant peaks at 9.1°, 11.7°, and 23.5° are consistent with higher order basal and in‐plane reflections of the V_2_CT_x_ phase [20, 34]. These peaks indicate increased interlayer spacing after selective etching of Al from V_2_AlC and the subsequent transformation of delaminated V_2_AlC into V_2_CT_x_ MXene, validating successful delamination. The sharp (002) peak indicates high crystallinity. Only a negligible peak remains from V_2_AlC around 2θ of 13.5°.

The hybrid materials (NF25 and NF50) with 25 and 50 wt.% V_2_CT_x_ exhibited notable changes relative to their pure components. The MXene (002) reflection is fully absent in both composites, indicating that the well‐ordered layer stacking of V_2_CT_x_ is disrupted during hydrothermal synthesis. This is attributed to oxidation of the MXene surface terminations and intercalation of the growing LDH phase between the MXene layers, both consistent with XPS evidence of mixed vanadium oxidation states discussed in the next paragraph. A broad (003) reflection persists in both composites at 10.09° (d_003_  =  8.76 Å, *c * =  26.28 Å), confirming retention of the LDH structure. The *a*‐parameter is unchanged in both pure NiFe‐LDH and the composites, demonstrating that MXene incorporation exclusively expands the interlayer spacing (Δ_003_  =  +0.16 Å) without altering the in‐plane brucite‐like layer structure. This selective *c*‐axis expansion may reflect partial accommodation of residual MXene surface functional groups within the LDH gallery. The (015) reflection, sensitive to *c*‐parameter changes, shifts from 40.46° in pure NiFe‐LDH to 38.50° in the composites (observed Δ2θ  =  −1.96°), consistent in direction with the expanded lattice; the magnitude of this shift is larger than predicted from the ideal hexagonal formula, which is expected for turbostratic LDHs where reflections with mixed (*hkl*) character are broadened and displaced by stacking disorder [28]. A weak reflection at 22.22° (d  =  4.00 Å), absent in pure NiFe‐LDH, is tentatively assigned to the V_2_CT_x_ (008) harmonic (theoretical d  =  3.995 Å, Δ  =  0.02° from the V_2_CT_x_ c‐parameter of 31.96 Å), suggesting that short‐range MXene stacking periodicity persists in the composites despite the complete loss of long‐range order. The region around 33–36° in both composites contains overlapping contributions from the LDH (012) reflection and the (311) plane of a minor NiFe_2_O_4_ spinel phase (reference d  =  2.514 Å); the two contributions cannot be fully resolved given the turbostratic peak broadening in this region. Spinel presence is confirmed unambiguously by a reflection at 63.20° (d  =  1.470 Å), which matches the spinel (440) plane to within Δd  =  0.004 Å of the reference value (1.474 Å, *a*  =  8.339 Å) [35] and is fully absent in pure NiFe‐LDH. The absence of isolated (311) and (400) reflections at 35.68° and 43.37° respectively confirms only minor spinel content. The growth of this minor spinel phase can be explained by two synergistic effects: (i) high‐surface‐area MXene nanosheets provide abundant nucleation sites favoring ordered crystalline phases over amorphous growth, and (ii) during hydrothermal synthesis, V_2_CT_x_ acts as a reducing agent whose oxidation from low‐valent carbide provides electrons that facilitate Fe_2_NiO_4_ spinel nucleation through transient Fe reduction [36].

#### 3.1.3 Transmission Electron Microscopy and Electron Energy Loss Spectroscopy

**FIGURE 3:**
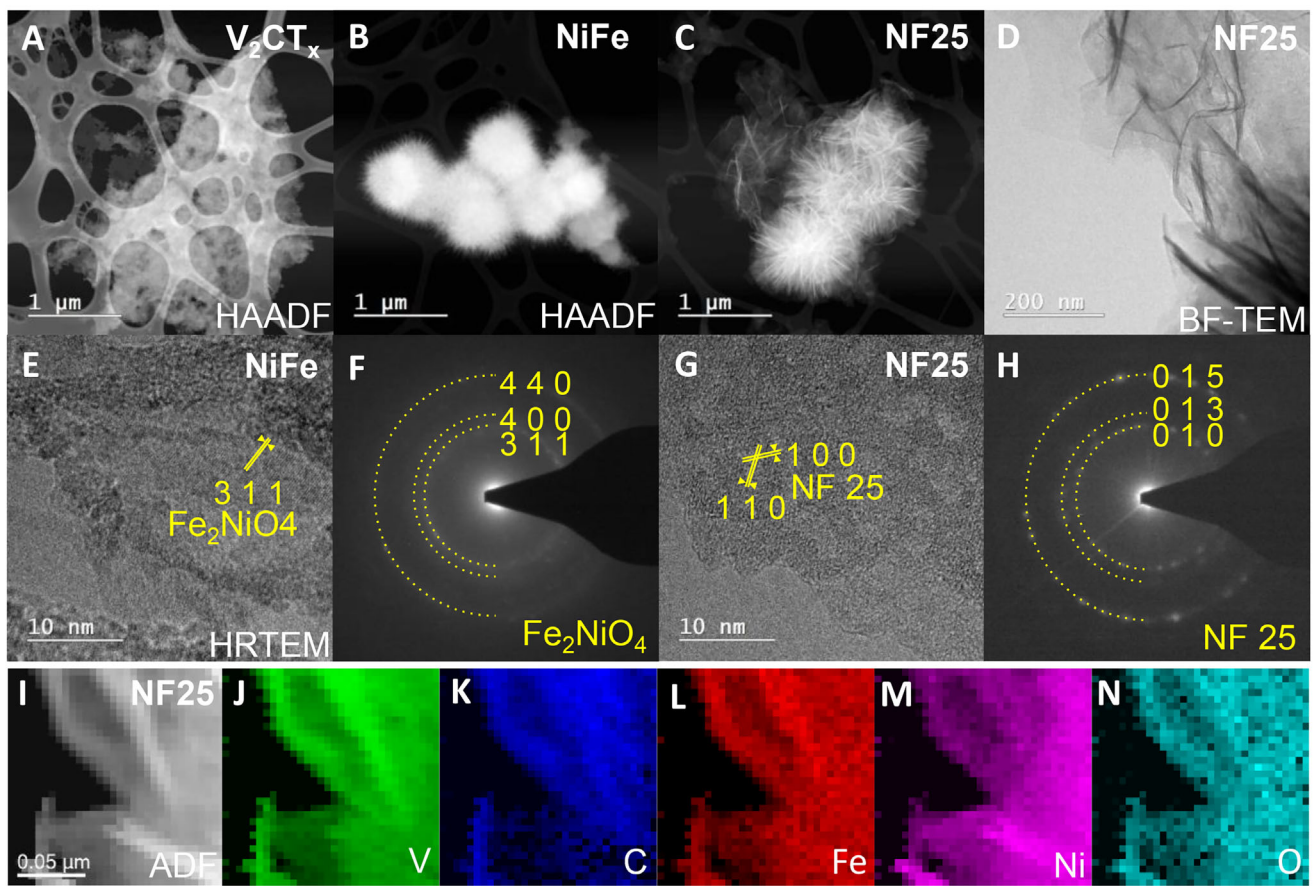
Transmission electron microscopy and EELS characterization of NiFe@V_2_CT_x_ composites. (A) HAADF‐STEM image of pure V_2_CT_x_ showing the characteristic open layered sheet network. (B) HAADF‐STEM image of pure NiFe showing compact urchin‐like aggregates. (C) HAADF‐STEM image of NF25. NiFe clusters are spatially anchored within the V_2_CT_x_ sheet scaffold. (D) TEM image of NF25 showing the homogeneous flake‐like composite structure and intimate contact between the two phases. (E,F) HRTEM image and SAED pattern of pure NiFe; lattice fringes and polycrystalline rings are indexed to Fe_2_NiO_4_ spinel (311), (400), (440). (G,H) HRTEM image and SAED pattern of NF25; lattice fringes in (G) are indexed to V_2_CT_x_ in‐plane (1 0 0) and ($\bar{1}$ 1 0) planes, indicating locally retained MXene sheet crystallinity, while diffuse SAED rings in (H) are indexed to LDH reflections (010), (013), (015) with no discrete MXene diffraction spots, confirming loss of V_2_CT_x_ long‐range stacking order. (I–N) EELS elemental maps of a NF25 composite region (scale bar 50 nm): (I) ADF‐STEM reference image, (J) vanadium, (K) carbon, (L) iron, (M) nickel, (N) oxygen.

Transmission electron microscopy (TEM) and high‐angle annular dark‐field scanning TEM (HAADF‐STEM) were performed on all synthesized materials to resolve the nanoscale structural changes and directly visualize the V_2_CT_x_‐NiFe interface (Figure 3 and Figures S4–S6). HAADF‐STEM confirms the morphological evolution observed by SEM: pure V_2_CT_x_ presents an open, porous layered sheet network (Figure 3A and Figure S4A–D), while pure NiFe consists of compact urchin‐like aggregates (Figure 3B and Figure S4J–M). In NF25, the urchin‐like NiFe morphology is retained but the clusters are spatially anchored within the V_2_CT_x_ sheet scaffold (Figure 3C and Figure S5B,C), indicating that the MXene surface acts as a nucleation template without fully suppressing the intrinsic NiFe growth habit. TEM imaging reveals NiFe and V_2_CT_x_ forming a homogenous flake‐like composite structure (Figure 3D and Figure S5A,D,E), confirming intimate physical contact between the two phases across multiple length scales. The same morphological characteristics are observed for NF50, where HAADF‐STEM and TEM imaging show an equivalent distribution of NiFe clusters across the V_2_CT_x_ scaffold at higher MXene loading (Figure S6A–F).

High resolution TEM (HRTEM) image and selected area electron diffraction (SAED) pattern provide complementary crystallographic evidence for phase assignment (Figure 3E–H and Figure S4E,F). Pure V_2_CT_x_ yields a sharp [0 0 1] zone‐axis SAED pattern with discrete diffraction spots indexed to the (0 $\bar{1}$0), (1 0 0), and ($\bar{1}$1 0) planes (Figure S4F), confirming single‐crystal quality and long‐range stacking order of the delaminated MXene. Pure NiFe displays clear lattice fringes indexed to the (3 1 1) plane of Fe_2_NiO_4_, corroborated by a SAED ring pattern matching the (3 1 1), (4 0 0), and (4 4 0) reflections of the spinel structure (Figure 3E,F), independently confirming the minor spinel phase identified by XRD. In both NF25 and NF50, HRTEM images at 10 nm resolution reveal a nanocrystalline texture (Figure 3G and Figure S6G) with lattice fringes indexed to the V_2_CT_x_ in‐plane (1 0 0) and ($\bar{1}$ 1 0) planes with a characteristic 120° inter‐fringe angle, consistent with the [0 0 1] zone axis of locally retained V_2_CT_x_ sheet crystallinity. However, the corresponding SAED patterns display only diffuse polycrystalline rings indexed to LDH reflections (010), (013), and (015), with a complete absence of discrete MXene diffraction spots (Figure 3H and Figure S6H), confirming that the long‐range periodic stacking order between V_2_CT_x_ layers is lost upon composite formation. Together, HRTEM and SAED reveal that locally crystalline V_2_CT_x_ sheets persist as individual 2D domains but lose their interlayer integrity. This is consistent with the turbostratic stacking disorder characteristic of hydrothermally synthesized LDHs [28] and directly corroborates the disappearance of the V_2_CT_x_ (002) reflection observed in XRD. Notably, this loss of MXene long‐range order is observed at both 25 and 50 wt.% V_2_CT_x_ loading, confirming it is an intrinsic consequence of hydrothermal synthesis rather than a loading‐dependent effect.

Electron energy loss spectroscopy (EELS) elemental mapping of NF25 and NF50 composite regions (Figure 3I–N and Figures S5F–Q and S6I–T) consistently shows that vanadium and carbon signals are spatially co‐localized within the MXene‐rich regions, while iron, nickel, and oxygen are concentrated in the adjacent LDH phase. Two independent regions were mapped for each composite, confirming the reproducibility of the elemental distribution (Figure S5F–Q for NF25; Figure S6I–T for NF50). Notably, the Fe, Ni, and O signals are detected within the V_2_CT_x_ sheet regions rather than being strictly confined to the phase boundary, indicating that the NiFe hydroxide phase penetrates between the MXene layer. This is consistent with LDH intercalation between V_2_CT_x_ layers and directly corroborates the c‐axis expansions observed by XRD, where the d_003_ spacing increases from 8.60 Å in pure NiFe‐LDH to 8.76 Å in the composites. The oxygen signal confined to the Ni/Fe region confirms the hydroxide/oxyhydroxide character of the NiFe phase [4], while its absence from the V/C‐rich carbide core is consistent with the partial, rather than complete, MXene oxidation observed by XPS and operando XANES [20]. The co‐existence of locally crystalline V_2_CT_x_ sheets, as evidenced by HRTEM, with intimately incorporated NiFe hydroxide species, reproduced across both loadings and multiple sample regions, establishes intimate atomic‐scale contact.

#### 3.1.4 X‐Ray Photoelectron Spectroscopy (XPS)

**FIGURE 4:**
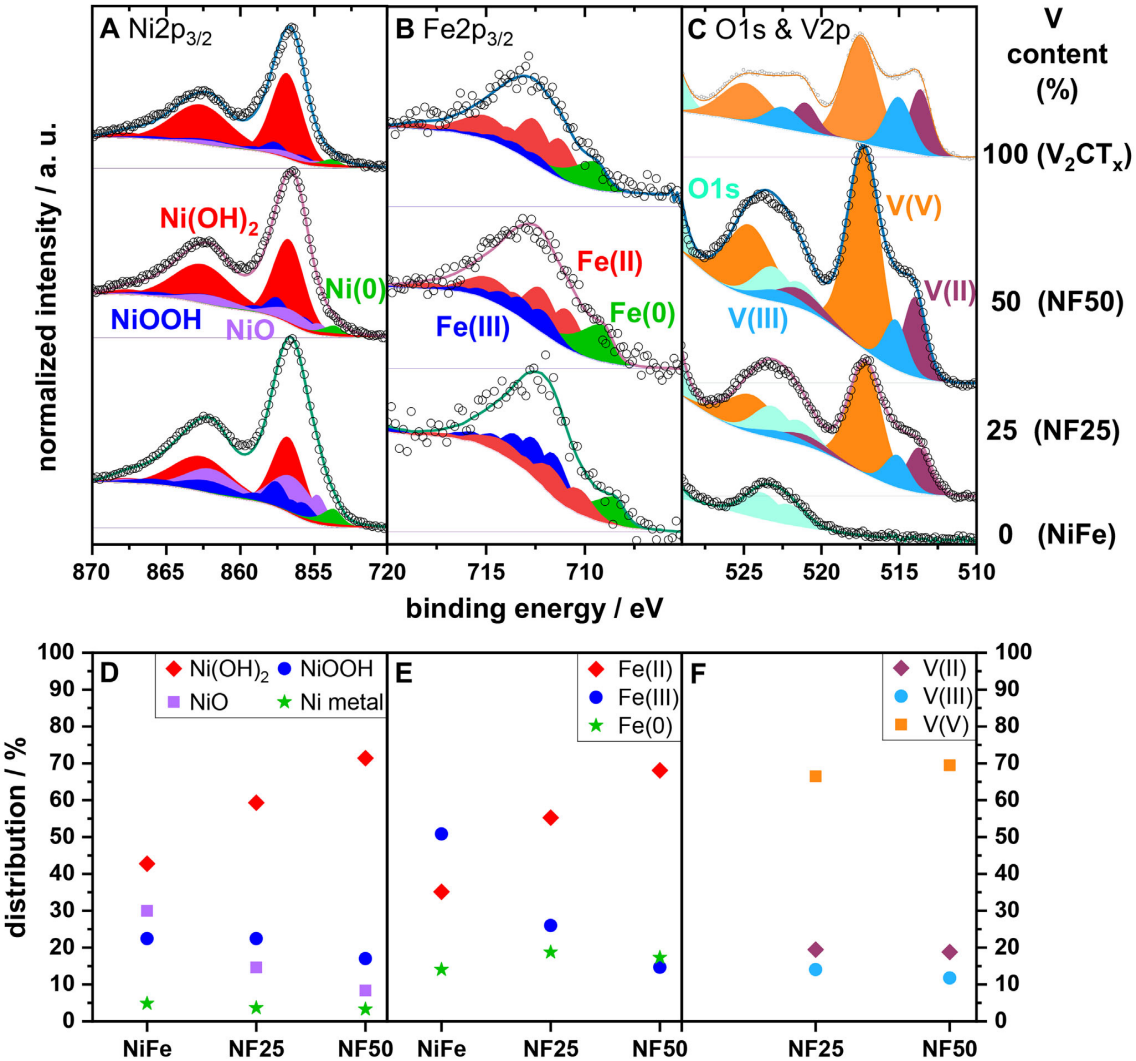
X‐ray photoelectron spectroscopy of NiFe@V_2_CT_x_ composites. (A–C) Deconvoluted peak fits of (A) Ni2p_3/2_, (B) Fe2p_3/2_, and (C) O1s + V2p; open circles represent experimental data and solid lines the fitted envelope and individual components. (D–F) Quantified oxidation state distributions (%) derived from peak integration for (D) nickel, (E) iron, and (F) vanadium.

The Ni2p, Fe2p, and O1s+V2p core levels of pristine NiFe, NF25, and NF50 with deconvoluted fits are shown in Figure 4A–C, raw O1s+V2p spectra are shown in Figure S7A, and quantified oxidation state distributions in Figure 4D–F.

Deconvolution of the Ni 2p spectra (Figure 4A,D) identifies four contributions: Ni(OH)_2_, NiOOH, NiO, and metallic Ni^0^. In pure NiFe, Ni(OH)_2_ accounts for 42.74% of the signal, consistent with the expected LDH structure, while NiO contributes 29.98% and NiOOH 22.44%. With increasing V_2_CT_x_ loading, Ni(OH)_2_ increases progressively to 59.33% in NF25 and 71.38% in NF50, while NiO decreases sharply to 14.60% and 8.35% respectively, indicating that V_2_CT_x_ incorporation stabilizes the Ni^2+^ hydroxide environment at the expense of the oxide phase. Notably, the calculated average nickel oxidation state remains essentially constant across the series at 2.13 (NiFe), 2.15 (NF25), and 2.10 (NF50), confirming that the overall Ni redox state is preserved despite the compositional redistribution. The modified Auger parameter of 1698.3 eV, Figure S7B is consistent with Ni(OH)_2_ across all samples [21], and metallic Ni^0^ remains a minor contribution (3.3–4.8%) throughout.

The Fe2p deconvolution (Figure 4B,E) reveals the most pronounced and chemically significant changes across the series. In pure NiFe, Fe(III) is the dominant species at 50.81%, with Fe(II) at 35.15%, yielding an average iron oxidation state of 2.23, consistent with a predominantly Fe^3+^ LDH environment with partial Fe^2+^ character. With increasing V_2_CT_x_ content, Fe(III) decreases dramatically to 26.01% (NF25) and 14.64% (NF50), while Fe(II) rises to 55.25% and 68.07% respectively, driving the average iron oxidation state down to 1.89 in NF25 and 1.80 in NF50. This systematic and loading‐dependent reduction of Fe^3+^ to Fe^2+^ provides direct quantitative evidence that V_2_CT_x_ acts as a reducing agent during hydrothermal synthesis, with low‐valent vanadium carbide donating electrons preferentially to iron rather than nickel, as evidenced by the near‐constant Ni average oxidation state contrasting with the sharp Fe reduction. This selective Fe reduction directly corroborates the formation of the Fe_2_NiO_4_ spinel phase identified by XRD (paragraph 3.1.3), in which iron occupies a mixed Fe^2+^/Fe^3+^ environment [36].

The V2p spectra (Figure 4C,F) show that vanadium is dominated by V(V) in both composites, accounting for 66.48% in NF25 and 69.47% in NF50, with low contributions from V(III) (∼12–14%) and V(II) (∼19%). This oxidation state distribution confirms substantial pre‐oxidation of V_2_CT_x_ during hydrothermal synthesis, prior to any electrochemical operation. The near‐identical vanadium distributions in NF25 and NF50 indicate that the degree of MXene oxidation is independent of loading and is an intrinsic consequence of the synthesis conditions. Taken together with the Fe reduction trend, the XPS data establish a coherent redox picture for the hydrothermal synthesis: V_2_CT_x_ oxidizes from low‐valent carbide (V^2+^/^3+^) to a V^5+^‐dominated mixed‐valence state while simultaneously reducing Fe^3^^+^ to Fe^2+^ in the forming LDH phase. XPS‐derived atomic ratios (Ni:Fe:V) of 95:5:0 for NiFe, 50:7:43 for NF25, and 34:5:61 for NF50 indicate smaller iron and larger vanadium incorporation compared to nominal values, consistent with partial incorporation of iron into the Fe_2_NiO_4_ spinel phase during synthesis.

### 3.2 Electrochemical Characterization

**FIGURE 5:**
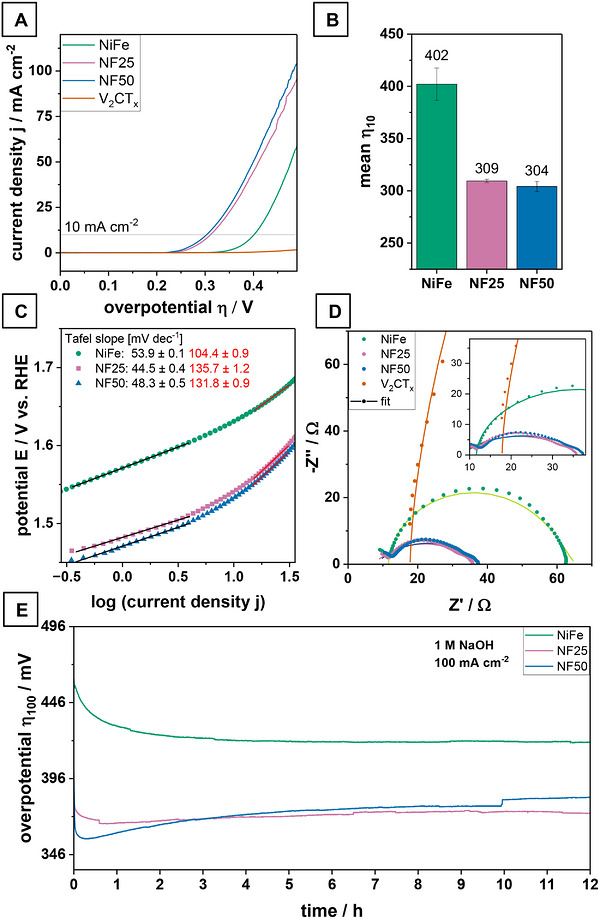
Electrochemical performance of NiFe@V_2_CT_x_ composites. (A) Linear sweep voltammograms recorded at 1 mV s^−1^ in 1.0 M NaOH electrolyte of pure NiFe, pure V_2_CT_x_ and materials NF25 and NF50, showing enhanced OER activity for composite materials. Current densities are normalized to geometric electrode area (0.0707 cm^2^). (B) overpotentials required to achieve at 10 mA cm^−2^ current density (η_10_), determined from at least three independent measurements per material. Error bars represent standard deviation. (C) Tafel plots of NiFe, NF25 and NF50, derived from LSV data. (D) Nyquist plots from electrochemical impedance spectroscopy at 1.65 V vs. RHE, with corresponding fits. (E) Chronopotentiometric stability testing at 100 mA cm^−2^ over 12 h for NiFe, NF25, and NF50. Overpotential values represent the potential vs. RHE required to maintain 100 mA cm^−2^ minus the thermodynamic potential (1.23 V).

The oxygen evolution reaction (OER) activity of the synthesized materials was evaluated via a suite of electrochemical measurements. Typical linear sweep voltammetry (LSV) curves of the pure NiFe, V_2_CT_x_, and the composite materials NiFe@25% V_2_CT_x_ (NF25) and NiFe@50% V_2_CT_x_ (NF50) are presented in Figure 5A, with the corresponding overpotentials required to achieve a current density of 10 mA cm^−2^ shown in Figure 5B. The composite materials demonstrated significantly enhanced OER activity compared to their individual components. The mean overpotentials (± standard deviation) required to reach 10 mA cm^−2^ (η10) were 402 ± 15 mV for pure NiFe, 309 ± 2 mV for NF25, and 304 ± 5 mV for NF50. The pure V_2_CT_x_ MXene did not reach 10 mA cm^−2^ within the overpotential range, indicating poor intrinsic OER activity. However, the incorporation of V_2_CT_x_ into the composite materials resulted in a substantial reduction in overpotential, with NF25 and NF50 showing improvements of 93 and 98 mV, respectively, compared to pure NiFe. This enhancement demonstrates the synergistic effect between the NiFe active phase and the V_2_CT_x_ MXene support.

To obtain insight into the OER mechanism and reaction kinetics, Tafel plots were constructed by plotting the potential (E) against the logarithmic current density log(j) and the Tafel slope was determined (Figure 5C). Interestingly, all materials exhibited two distinct linear regions with different slopes, indicating a change in the rate‐determining step across different current density ranges. In the low current density region, pure NiFe showed a Tafel slope of 53.9 ± 0.1 mV dec^−1^, while the composite materials NF25 and NF50 displayed slopes of 44.5 ± 0.4 and 48.3 ± 0.5 mV dec^−1^, respectively. These values approach the theoretical 40 mV dec^−1^ for ^−^OOH intermediate formation as rate‐determining step [37]. At higher current densities, all materials showed significantly larger Tafel slopes: 104.4 ± 0.9 mV dec^−1^ for NiFe, 135.7 ± 1.2 mV dec^−1^ for NF25, and 131.8 ± 0.9 mV dec^−1^ for NF50; suggesting that the composites develop more significant mass transport limitations at elevated current densities despite their enhanced intrinsic activity. This may be attributed to the altered GDE morphology in the composites: the disruption of the urchin‐like NiFe microstructure by V_2_CT_x_ incorporation, as observed by SEM (Figure 2), creates a more compact, sheet‐like catalyst layer that could restrict gas evolution and electrolyte access at high current densities. Notably, this mass‐transport penalty does not affect device‐level performance significantly, as the composite electrodes achieve lower cell voltages than pure NiFe throughout the operating range of the AEM electrolyzer (Figure 8B), where the electrode configuration and high‐surface‐area gas diffusion layer (GDL) substrate alleviate such limitations [36]. The lower Tafel slopes of the composite materials in the low current density region demonstrate improved reaction kinetics, supporting the enhanced OER activity observed in the LSV measurements.

To gain deeper insight into the charge transfer kinetics, potentiostatic electrochemical impedance spectroscopy (EIS) was conducted in the OER region (Figure S9). The charge transfer resistance values determined at 1.65 V vs. RHE were significantly different among the materials tested (Figure 5D). Pure V_2_CT_x_ exhibited the highest charge transfer resistance (R_ct_) of 1741 Ω, demonstrating its poor electrocatalytic activity for OER, which is consistent with the LSV results where it failed to reach 10 mA cm^−2^. Pure NiFe showed an R_ct_ of 53.9 Ω, indicating moderate charge transfer kinetics. Remarkably, both composite materials NF25 and NF50 demonstrated nearly identical and substantially reduced R_ct_ values of 29.3 and 30.2 Ω respectively, representing an approximate 50% decrease compared to pure NiFe. This significant reduction in charge transfer resistance indicates enhanced electron transfer kinetics at the electrode‐electrolyte interface, which directly correlates with the improved OER activity observed in the LSV measurements. The similar R_ct_ values for NF25 and NF50 suggest that the optimal electronic conductivity enhancement is achieved at relatively low V_2_CT_x_ loadings, with further increases in MXene content not providing additional benefits to the charge transfer process.

Long‐term stability tests were conducted using chronopotentiometry at a constant current density of 100 mA cm^−2^ (Figure 5E and Figure S8). Pure NiFe demonstrated improved performance over time, with the overpotential decreasing from an initial 460 to 418 mV, indicating catalyst activation during operation. This activation behavior is commonly observed in transition metal‐based OER catalysts and is typically attributed to surface reconstruction and optimization of active sites. In contrast, both composite materials showed slight increases in overpotential during the stability test. Pure V_2_CT_x_, shown in Figure S8, displayed the poorest initial performance with an overpotential of 526 mV, which decreased to 466 mV over time, though this improvement was insufficient to compete with the composite materials. The characteristic initial overpotential hump observed for pure V_2_CT_x_, peaking within the first hours before gradually declining, is consistent with the progressive oxidation of the V_2_CT_x_ carbide under anodic OER conditions, a transformation directly observed by operando XANES, where the V K‐edge shifts to higher energies indicative of V^2+^/^3+^ → V^3+^/^4+^/^5+^ oxidation (Paragraph 3.3). The subsequent overpotential decrease reflects the formation of electrochemically active mixed‐valence vanadium oxide species, which partially compensate for the loss of the intrinsic MXene conductivity. However, in the absence of NiFe active sites, the final overpotential remains substantially higher than that of the composite materials, confirming that the vanadium oxide species alone are insufficient for competitive OER activity. Despite the slight overpotential increases observed for the composite materials, both NF25 and NF50 maintained significantly lower overpotentials throughout the stability test compared to pure NiFe, demonstrating superior catalytic performance. The better stability of NF25 compared to NF50 suggests that a moderate V_2_CT_x_ loading (25%) provides the optimal balance between enhanced activity and long‐term stability.

To better understand the relationship between surface properties and catalytic performance, the electrochemically active surface area (ECSA) of the pure materials, and composites were determined using the electrical double‐layer capacitance *C_dl_* derived from cyclic voltammetry at varying scan rates in the non‐faradaic potential region (Figure S10). ECSA measurements show variability typical of MXene composites, with mean values (± SD) of 26.5 ± 10.7 cm^2^ (NiFe), 27.7 ± 10.2 cm^2^ (NF25), 19.8 ± 7.7 cm^2^ (NF50), and 27.1 ± 7.1 cm^2^ (V_2_CT_x_) (Figure S10F). The high variability (±37%–55%) precludes precise quantitative comparison between materials. Nevertheless, the similar mean ECSA values across NiFe, NF25, and V_2_CT_x_, and the only modestly lower value for NF50, confirm that the substantially lower overpotential of NF25 (93 mV improvement) cannot be attributed to a proportional increase in active surface area, and instead reflects enhanced intrinsic turnover activity consistent with V_2_CT_x_‐mediated electronic structure modification revealed by operando XAS.

### 3.3 Operando X‐Ray Absorption Spectroscopy

#### 3.3.1 X‐Ray Absorption Near‐Edge Structure (XANES) Analysis

**FIGURE 6:**
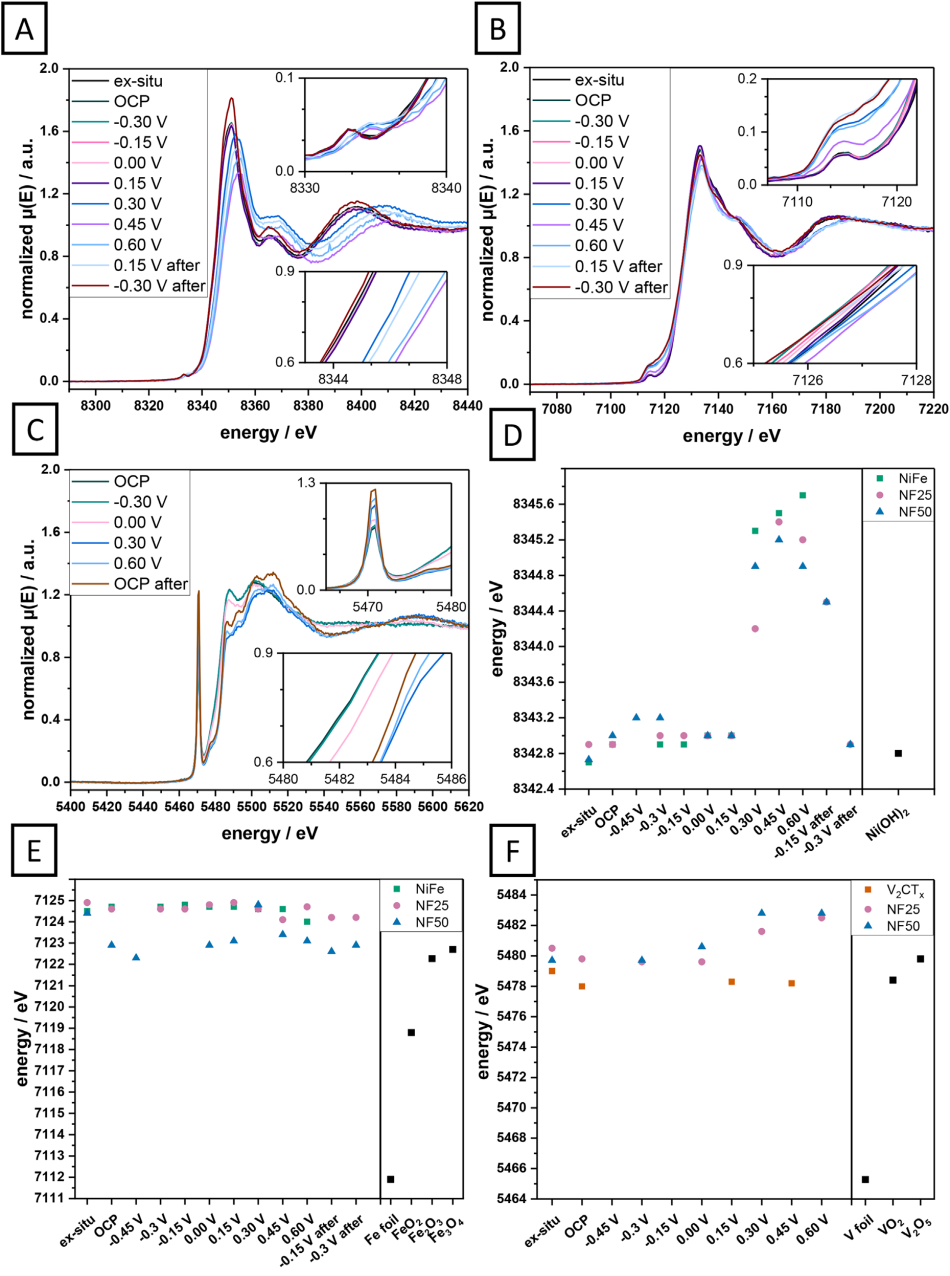
Operando X‐ray absorption near edge spectroscopy (XANES) and extended X‐ray absorption fine structure analysis (EXAFS). XANES spectra showing the (A) Ni, (B) Fe, and (C) V K‐edge and pre‐edge of NF25 under various applied OER overpotentials. (D) Ni edge energies of NiFe, NF25, and NF50 plotted against the applied overpotential and ex‐situ energy of Ni(OH)_2_ reference. (E) Fe edge energies of NiFe, NF25, and NF50 plotted against the applied overpotential and ex‐situ energies of Fe foil, FeO_2_, Fe_2_O_3_, and Fe_3_O_4_ references. (F) V edge energies of V_2_CT_x_, NF25, and NF50 plotted against the applied overpotential and V foil, VO_2_, and V_2_O_5_ references. All measurements were performed in 1.0 M NaOH electrolyte using a three‐electrode configuration.

Operando X‐ray absorption spectroscopy (XAS) was conducted to understand the atomic‐scale mechanisms underlying the enhanced OER performance in NiFe@V_2_CT_x_ composites observed in Figure 5. Sequential analysis of nickel, iron and vanadium K‐edges demonstrates electronic coupling between MXene and NiFe catalyst that contributes to the observed catalyst enhancement. Figure 6A–C displays the XANES spectra of the Ni, Fe, and V K‐edges of the best performing catalyst NF25, respectively, as well as the evolution of the energy positions of all observed materials (Figure 6D–F). XANES spectra of pure NiFe, V_2_CT_x_ and NF50 are shown in Figure S11.

The Ni K‐edge XANES reveals modified nickel redox behavior in V_2_CT_x_‐containing composites during the OER (Figure 6A–F and Figure S11D–F). The pure NiFe catalyst exhibits a systematic positive absorption edge shift from 8342.9 eV (OCP) to 8345.7 eV (0.6 V) with increasing applied potential, Figure S11A. This behavior is consistent with the well‐established Ni^2+^→ Ni^3+^ oxidation that activates NiFe catalysts for OER [6, 7, 25, 38, 39]. The reversible nature of this shift is consistent with the reversibility of the Ni(II) → Ni(III) transition during potential cycling.

Given that the catalytic activity increases with the addition of V_2_CT_x_, Ni edge shifts toward higher oxidation states would be expected in the composite materials if the activity increase would result only from structural reconstruction. Therefore, the suppressed nickel oxidation cannot be attributed to decreased electrochemical accessibility of active sites or an increase of surface defects, as ECSA measurements confirm comparable surface areas between NF25 and NF50. Instead, this electronic modulation indicates V_2_CT_x_‐mediated electronic buffering (mechanism through V K‐edge analysis) that influences nickel oxidation states during OER. The difference of the absorption edge in our NiFe materials in comparison to the pure nickel hydroxide reference for the same potential is consistent with the early promotion of the nickel oxidation states in the NiFe material due to the presence of iron [38, 39]. This promotion by iron doping, a known phenomenon in literature, is also correlated with a geometrical change of the catalyst structure indicated by a simultaneous pre‐edge region change in Figure 6B and Figure S11B which corresponds to the formation of nickel‐iron oxyhydroxide. This phase can be determined as the active phase for OER in this work, however the modulation of its activity by the addition of MXene is not correlated with the Ni and Fe‐edge and pre‐edge positions/shapes.

Fe K‐edge XANES analysis reveals that iron maintains its essential catalytic role while showing enhanced coordination stability in V_2_CT_x_‐containing composites (Figure 6B and Figure S11E). The absorption edge position remains relatively constant at 7124.5 ± 0.3 eV across all potentials, showing that iron predominantly exists as Fe^3+^ throughout the catalytic operation. It should be noted, however, that the constancy of the absorption edge position and the observed pre‐edge changes are not entirely equivalent indicators, the former primarily reports the average Fe oxidation state, while the latter reflects changes in the local coordination symmetry. The systematic pre‐edge intensity increases under OER conditions are therefore most accurately interpreted as evidence of a structural transformation from octahedral to distorted octahedral or tetrahedral coordination characteristic of FeOOH, rather than as direct evidence of Fe oxidation state change. This distinction is consistent with the established role of Fe^3^^+^ as a site whose coordination environment evolves during the LDH‐to‐oxyhydroxide transition without a net change in formal oxidation state [40], and with the more pronounced pre‐edge variations observed in NF25 and NF50 compared to pure NiFe, which may reflect a modified Fe local environment induced by V_2_CT_x_ interaction.

The most striking findings emerge from the analysis of the V K‐edge XANES, which reveals dramatic structural transformation of V_2_CT_x_ under OER conditions (Figure S11C). Pure V_2_CT_x_ MXene initially displays characteristic features of vanadium carbide: edge position at 5478.0 eV, a weak pre‐edge at 5470 eV (pre‐edge to main edge ratio ∼0.2) and a strong first oscillation at 5488 eV [41, 42, 43]. NF25 and NF50 composites initially show slightly higher edge positions than the pure V_2_CT_x_ (Figure 6F), revealing partial vanadium oxidation during hydrothermal synthesis. Upon applying 0.45 V, the spectra undergo systematic changes that intensify at higher potentials (Figure 6C and Figure S11C,F). At 0.6 V NF25 and NF50 show edge positions of 5483.1 and 5482.5 eV, respectively. These edge positions, exceeding even the V_2_O_5_ reference, provide strong evidence for V^5+^ formation. The pre‐edge to main edge ratio increases from 0.2 to 1.0 (Figure 6C), indicating a transition from octahedral VC_6_ to distorted VO_6_ coordination [41]. This increase in 1s → 3d transition probability reflects loss of centrosymmetry upon oxidation, consistent with V^5+^ in distorted coordination. However, a distinct shoulder emerges at 5468 eV, and the first oscillation, the white line, at 5488 eV diminishes while a second oscillation at 5510 eV intensifies, indicating a mixed valence state rather than pure V^5+^ [41, 42, 43]. This mixed state would provide an even broader redox window for electronic buffering, with multiple electron transfer pathways available. Following this analysis, one can see from the ex‐situ spectrum of the 50% composite in Figure S11F that the vanadium contained in the catalysts presents a mix of states. Its pre‐edge has a ratio close to 1:1 with the edge intensity and a strong second oscillation, while conserving a very defined first oscillation at 5488 eV. This mix indicates either the presence of vanadium oxide in the pre‐catalyst, or a partial oxidation of the MXene with the functionalization of its surface during pre‐catalyst synthesis. Either one being true, a certain observation is the oxidation of a significant part of the remaining V_2_CT_x_ under OER conditions as shown by the drop of the first oscillation intensity, the increase in pre‐edge intensity, and the appearance of the second pre‐edge peak shown in Figure S11F.

Given that the addition of MXene is responsible for higher catalytic activity compared to the pure material, this vanadium transition could be an enhancing factor through a doping effect. The possibility of a charge transfer enhancement through the conservation of the MXene metallic plane order after the structural changes shouldn't however be discarded: a recent OER study by Najafi et al. showed that the transformation of 2D vanadium selenide allowed the formation of metallic vanadium oxide [44].

#### 3.3.2 Extended X‐Ray Absorption Fine Structure (EXAFS) Analysis

**FIGURE 7:**
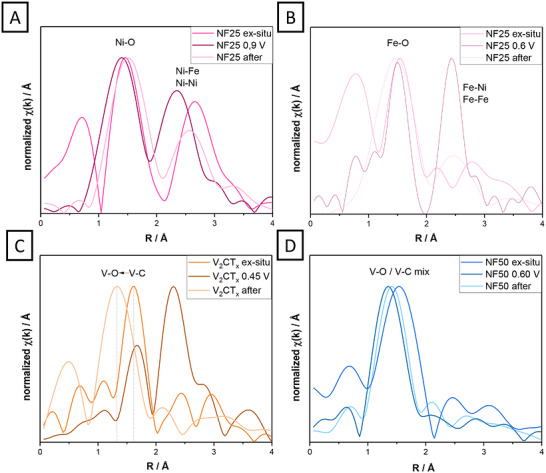
(A,B) Fourier‐transformed EXAFS spectra showing (A) metal‐oxygen and (B) coordination changes in NF25 during OER operation for nickel and iron edges, respectively. Progressive bond length contractions indicate LDH to γ‐oxyhydroxide transformation. (C,D) Vanadium coordination environment evolution in (C) pure V_2_CT_x_ and (D) NF50, demonstrating V─C to V─O bond transformation. All fits used in physical interpretation meet standard criteria (R‐factor < 0.02). Three excluded entries are flagged in Table S2.

EXAFS analysis was performed on the different absorption edges to determine coordination environment changes during electrochemical operation, Figure 7 (ex situ spectra shown in Figure S12).

The simulation of diffusion paths requires a precise crystalline structure which cannot be straightforwardly achieved with the present catalysts: the Wyckoff positions of nickel, iron, and hydroxyl groups vary in the NiFe catalyst, and similar issues arise for the vanadium coordination environment due to the coexistence of V─O and V─C bonds. As a consequence, a full structural fit could not be achieved, and first shell quantitative fits were performed following established protocols for operando XAS analysis. Fits used in physical interpretation meet standard criteria of R‐factor < 0.02 and positive σ^2^ values [26], however three entries with unphysical amplitude parameters are flagged in Table S2 and excluded from all interpretation. The V_2_CT_x_ post‐OER exclusion is attributed to signal attenuation following vanadium dissolution, while the NF50 high‐potential entries reflect structural heterogeneity in the mixed V─C/V─O coordination environment. No physical conclusions in this work depend on these excluded entries. Given the complexity of a multi‐component system, the fitted amplitude and energy shift parameters carry high uncertainties and are reported in Table S2 for completeness only, physical interpretation is limited to the bond distances and R‐factors. Reported metal‐oxygen (M─O) bond length analysis reveals systematic structural evolution consistent with transformation into oxyhydroxide structures (Figure 7A,B and Figure S11F).

Under applied potential, The Ni─O bond contracts from 2.035 Å at OCP to 1.935 Å at 0.6 V in NF25 (Figure 7A). This 0.1 Å contraction matches literature values for hydroxide to γ‐oxyhydroxide transformation, demonstrating activation of the NiFe catalyst [45, 46]. These structural changes show different reversibility behavior in composites compared to pure NiFe, suggesting that V_2_CT_x_ interaction influences the structural dynamics of the NiFe phase during electrochemical cycling.

The most significant structural changes occur in vanadium coordination (Figure 7D). Pure V_2_CT_x_ shows characteristic V─C bonds at 2.027 Å, but under OER conditions, these undergo transformation to V─O bonds at 1.841 Å—a substantial 0.186 Å contraction. This bond length change provides structural evidence for the vanadium oxidation observed by XANES and confirms the formation of vanadium oxide coordination environments [34, 41]. The short V─O distances fall within the range reported for vanadium oxides, while VO_2_ and V_2_O_3_ typically show V─O distances of 1.9–2.0 Å [41]. Our observed value, combined with the high edge energy, suggest predominantly V^5+^ character with possible V^3+^/V^4+^ contributions.

In comparison, composite materials show no evidence of significant variations in the vanadium first coordination shell under OER conditions, with a consistent radial distance ranging from 2.08 to 2.05 Å throughout the electrochemical operation (Figure 7D). This structural stability of the vanadium environment in the composites, despite the XANES‐evidenced oxidation state changes, indicates that the V_2_CT_x_ sheets in the composite retain an overall stable V(C,O)_6_ coordination sphere, consistent with the formation of V‐O‐M bridges that constrain the local vanadium structure while enabling electronic communication with the NiFe active sites.

Combining XANES edge positions with EXAFS bond lengths reveals a two‐stage transformation with distinct implications for catalytic performance. During hydrothermal synthesis and initial OER operation (‐0.30 – 0.45 V vs. RHE), V_2_CT_x_ undergoes progressive oxidation from low‐valent carbide (V^2+^/V^3+^, edge position 5478 eV) to mixed‐valence V^3+^/V^4+^/V^5+^ oxide species (edge reaching 5483 eV at 0.6 V vs. RHE). For pure V_2_CT_x_, EXAFS confirms V─C (2.027 Å) to V─O (1.841 Å) bond transformation with corresponding VC_6_ to VO_6_ coordination change. In the composites the average conservation of the vanadium first coordination shell distance (2.08–2.05 Å) indicates an overall structural stability of the vanadium environment during oxidation, consistent with the formation of stable V(C,O)_6_ coordination sphere.

This transformation creates V‐O‐M bridges that provide structural pathways for electronic communication between vanadium and NiFe. Once established, the mixed‐valence V^3+^/V^4+^/V^5+^ manifold creates a broad redox window that can dynamically modulate NiFe oxidation states through electron transfer. When nickel tends toward detrimental Ni^4+^ over‐oxidation, the vanadium can redistribute electrons through V‐O‐M bridges, stabilizing nickel in the optimal Ni^2+^/^3+^ state. This electronic modulation is evidenced by suppressed Ni K‐edge shifts in composites (2.3 eV) compared to pure NiFe (2.8 eV) under identical electrochemical conditions. Our operando measurements capture Stage 1 (formation of the active system) during progressive oxidation, while the enhanced electrochemical performance—reduced overpotentials (93‐98 mV improvement), lower charge‐transfer resistance (50% reduction), and improved stability—provides evidence for Stage 2 operational benefits. During the period of active vanadium presence, this electronic coupling prevents Ni over‐oxidation while preserving essential Fe^3+^ sites for O─O bond formation, an effect that is progressively attenuated as vanadium dissolves under extended alkaline polarization, as discussed in Paragraph 3.5. While V‐O‐M bridges identified by EXAFS demonstrate covalent interaction between vanadium and the NiFe matrix during OER operation, post‐mortem characterization by ICP‐OES, EDX, and XPS confirm that these interactions do not suppress vanadium dissolution under prolonged alkaline polarization. Quantification of absolute vanadium losses confirms this: ICP‐OES analysis of the 1 L anolyte after 144 h confirms absolute vanadium losses of 2.67 mg (NF25) and 4.90 mg (NF50), consistent with the near‐complete bulk depletion confirmed by post‐mortem EDX and the undetectable surface vanadium (confirmed by XPS). Consistent with Pourbaix thermodynamics, the vanadium oxide species formed during OER are not infinitely stable at pH 14. The V‐O‐M coupling observed by operando XAS is therefore understood as a transient but mechanistically significant interaction that modulates NiFe oxidation state dynamics during the period of vanadium presence. The resulting structural reorganization of the NiFe phase provides a lasting synthetic legacy that sustains the activity advantage even after the vanadium leaching.

Recent studies have reported MXene oxidation during electrochemistry [12], and our post‐mortem data confirm that also V_2_CT_x_ ultimately dissolves under extended alkaline OER conditions, as reported by others. Rather than simply challenging this picture, our work establishes a more nuanced design principle: even transient V‐O‐M coupling during the early period of operation is sufficient to restructure the NiFe phase in ways that provide durable activity enhancement. This reframes V_2_CT_x_ from active permanent mediator to sacrificial electronic modifier. A concept with broad implications for MXene‐composite catalyst design.

### 3.4 AEM Electrolyzer Device Measurements

**FIGURE 8:**
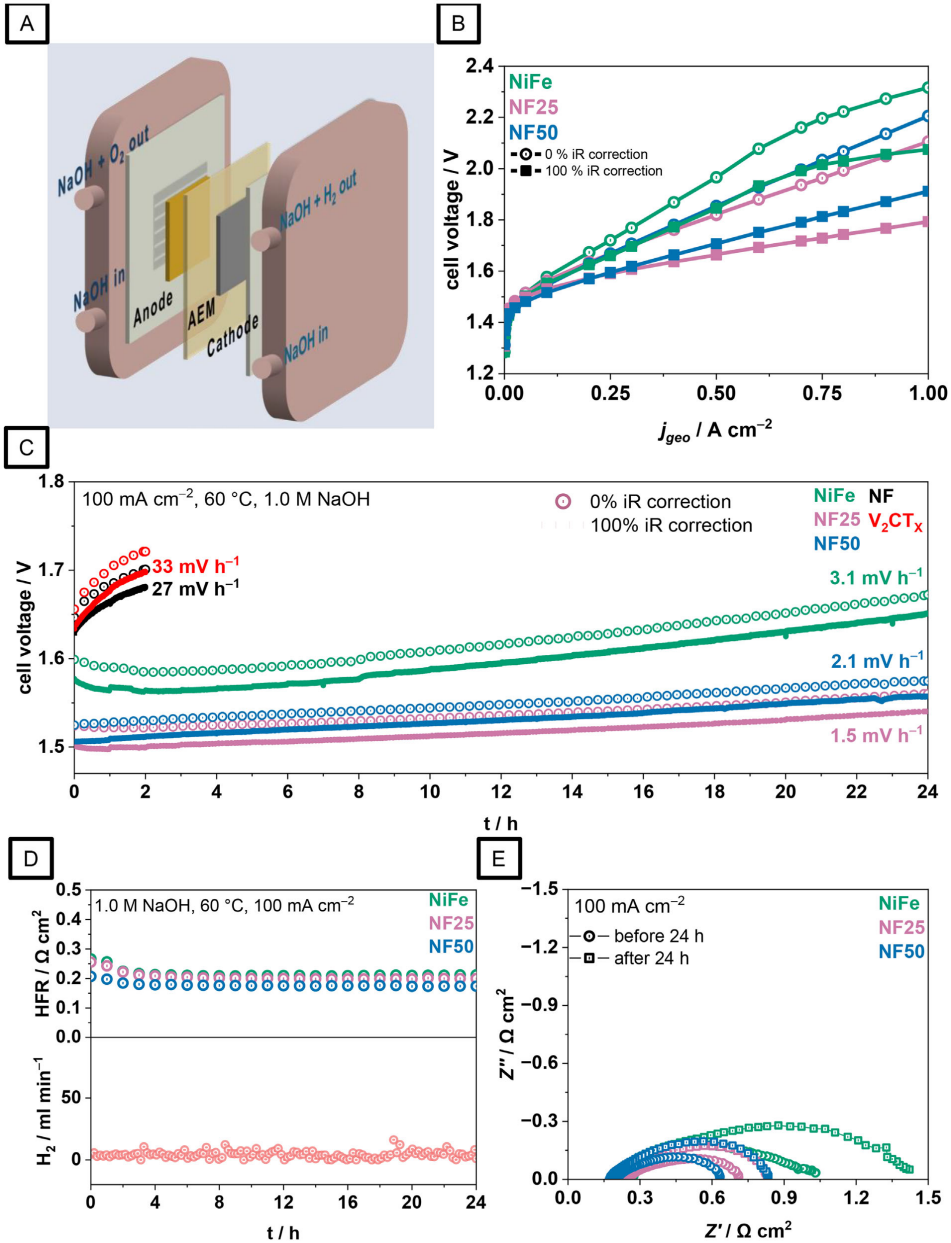
Evaluation of NiFe, NF25 and NF50 catalysts in a zero‐gap AEM electrolyzer. (A) Schematic of the AEM electrolyzer configuration, (B) Steady‐state polarization curves, (C) Chronopotentiometry at 100 mA cm^−2^ over 24 h, (D) High‐frequency resistance (HFR) and H_2_ production rate at 100 mA cm^−2^ during 24 h operation, and (E) galvanostatic electrochemical impedance spectroscopy (GEIS) at 100 mA cm^−2^ before and after stability testing. All measurements were performed in 1.0 M NaOH at 60°C.

To demonstrate the practical viability of the stable anode catalysts, NF25 and NF50 for electrochemical H_2_ production, zero‐gap anion‐exchange membrane (AEM) electrolyzer tests were performed at 60°C. A schematic of the electrolyzer is shown in Figure 8A. The pure NiFe, V_2_CT_x_ and nickel felt (NF) were also evaluated as anodes for comparison, with Pt/C on NF serving as the cathode in all tests. Steady‐state *I–V* curves for NiFe, NF25, and NF50 are presented in Figure 8B. At a low current density of 10 mA cm^−2^, NiFe, NF25, and NF50 exhibit similar cell voltages. However, at 250 mA cm^−2^, the V_2_CT_X_‐containing NiFe catalysts (NF25 and NF50) deliver lower cell voltages of about 1.59 V, 70 mV below that of bare NiFe. The performance advantage widens substantially at higher current densities. At 500 mA cm^−^^2^, NF25 and NF50 deliver iR‐corrected cell voltages of 1.663 and 1.707 V respectively, representing advantages of 182 and 138 mV over pure NiFe (1.845 V), more than double the separation observed at 250 mA cm^−2^. At 1000 mA cm^−2^, NF25 and NF50 achieve 1.793 and 1.912 V respectively, compared to 2.075 V for pure NiFe, corresponding to advantages of 282 and 163 mV, Figure 8B. This progressive widening of the voltage gap with increasing current density indicates that the electronic modification imparted by V_2_CT_x_ during synthesis alleviates the mass‐transport and conductivity limitations that become increasingly rate‐limiting at industrial current densities, consistent with the reduced charge‐transfer resistance observed by GEIS (Figure 8E). Notably, while NF25 outperforms NF50 in the fresh‐electrode *I*–*V* curve at 1000 mA cm^−2^ (1.793 V vs 1.912 V), NF50 achieves a lower absolute cell voltage plateau during the 1000 mA cm^−2^ stability phase (2.28 V vs 2.36 V for NF25, Section 3.5), suggesting that the higher V_2_CT_x_ loading drives more extensive structural reorganization of the NiFe phase during early operation, yielding a more active, albeit less kinetically stable, electrode at the highest current densities.

This performance is competitive with recently reported MXene‐based catalysts in AEM electrolyzers (detailed comparison in Table 1). This indicates that V_2_CT_X_ MXene enhances the intrinsic activity of NiFe. Catalyst stability under prolonged operation was assessed by running the electrolyzer at 100 mA cm^−2^ for 24 h at 60°C, with continuous H_2_ production recorded (Figure 8C). Initial cell voltages (without *iR* correction) were 1.60 V for NiFe, 80 mV higher than NF25 and NF50. Over 24 h, the NiFe voltage rose gradually to 1.65 V, corresponding to a degradation rate of 3.1 mV h^−1^. In contrast, NF25 and NF50 exhibited slower degradation (1.5 mV h^−1^ and 2.1 mV h^−1^, respectively), with NF25 showing a 50% lower voltage drift than NiFe. These results confirm that V_2_CT_x_ loaded NiFe provides more robust OER active sites, as observed in three‐electrode tests and translates to improved electrolyzer performance. The enhanced activity and stability of NF25 highlight the importance of optimal V_2_CT_X_ loading for long‐term operation. For further comparison, bare NF and V_2_CT_x_ loaded NF were tested as anodes, yielding cell voltages 140 mV higher than V_2_CT_x_ loaded NiFe composites, with rapid degradation rates of 27 and 33 mV h^−1^, respectively, leading to failure within few hours. The initial superior performance and lower degradation rate are attributed to V‐mediated electronic buffering during the early operation period, with the sustained activity advantage afterward attributable to the structural legacy of V_2_CT_x_ incorporation.

**TABLE 1:** Performance comparison of MXene‐based anode catalysts in AEM electrolyzers.

Anode catalyst	Cathode catalyst	Membrane	Electrolyte	Cell Voltages at 100 mA cm^−2^		References
				0 h	24 h	
NF25 (NiFe@25%V_2_CT_x_)	Pt/C	A80	1.0 M NaOH, 60°C	1.50 V	1.54 V	This work
NF50 (NiFe@50%V_2_CT_x_)	Pt/C	A80	1.0 M NaOH, 60°C	1.50 V	1.56 V	
CoFe@V_1.8_CT_x_ (CFVv75)	Pt/C	FAA‐3‐50	1.0 M NaOH, 60°C	1.48 V	1.63 V	[47]
Co@Mo_2_CT_x_	PtRu/C	Xion Pention‐72‐15CL	1.0 M KOH, 80°C	1.58 V		[48]
(Ni,Fe)S_2_@Ti_3_C_2_T_x_	Raney Ni	FAA‐3‐PK‐130	1.0 M KOH, 70°C	1.6 V		[49]
CoMnO_3_/CoMn_2_O_4_‐Ti_3_C_2_T_x_	Pt/C	FAA‐3‐50	1.0 M KOH, 60°C	1.58 V		[50]
CoZnCr@Mo_2_TiC_2_T_x_	CoZnCr@Mo_2_TiC_2_	—	1.0 M KOH, 60°C	1.55 V	1.55 V	[51]
RuO_2_	Ru‐RuP/V_2_CT_x_	X37‐50 Grade T	1.0 M KOH, 60°C	1.50 V		[52]
NiFe‐LDH/Ti_3_C_2_T_x_	NiFe‐LDH/Ti_3_C_2_	—	1.0 M KOH, RT	1.75 V	1.75 V	[53]

Additionally, the NF25 catalyst is more stable than our CoFe@V_1.8_CT_x_ catalyst from a previous study [47]. High‐frequency resistance (HFR) measurements during durability tests primarily reflect the ionic resistance of the membrane (Figure 8D). Across all catalysts, HFR remained below 50 mΩ cm^2^ and was stable throughout, validating the robustness of the membrane and electrolyzer assembly. Cathode H_2_ production rates ranged from 1–8 mL min^−1^ (mean value is 4 mL min^−1^), with variability likely arising from intermittent bubble accumulation and release.

Nyquist plots from galvanostatic electrochemical impedance spectroscopy (GEIS) before and after 24 h operation are shown in Figure 8E. NiFe displayed higher charge‐transfer resistance (R_ct_) than the MXene‐NiFe composites, with a substantial increase post‐durability. By contrast, NF25 and NF50 showed only minimal R_ct_ rises after 24 h. These electrochemical impedance spectroscopy (EIS) trends align with three‐electrode measurements, illustrating that V_2_CT_x_ MXene modulates the NiFe coordination environment. This modulation preserves NiOOH and Fe^3+^ active sites under anodic potentials, thereby sustaining lower charge transfer resistance and long‐term stability for the OER at anode in Zero‐gap AEM electrolyzers.

These findings further support enhanced electrolyzer performance, as evidenced by the stability tests, where the NF25 catalyst achieves the highest cell efficiency of 83%. This outperforms NF50 (82%) and the benchmark NiFe catalyst (78%), reflecting the benefits of V incorporation from V_2_CT_x_ MXene in stabilizing the active sites and minimizing overpotentials during prolonged operation. Detailed values for energy consumption, electrolysis power and cell efficiency of NiFe, NF25, and NF50 are provided in Table S3.

To contextualize these results within the field, Table 1 compares NF25 and NF50 performance against recently reported MXene‐based catalysts in AEM electrolyzers. While our three‐electrode overpotentials (304–309 mV) are higher than some reports using nickel foam substrates, this work uniquely combines (i) operando spectroscopic mechanistic insight into V_2_CT_x_ transformation, (ii) competitive cell voltages of 1.50 V at 100 mA cm^−2^, and (iii) superior long‐term stability with the lowest voltage degradation rate (1.5 mV h^−1^) among V_2_CT_x_‐based systems. These results validate that controlled MXene oxidation can provide a viable strategy for durable AEM water electrolysis.

### 3.5 Post‐Mortem Characterization

To probe durability beyond the initial 24 h testing period, NF25 and NF50 anodes were subjected to 144 h of continuous chronopotentiometric testing in the AEM electrolyzer, comprising 72 h at 500 mA cm^−2^ followed by 72 h at 1000 mA cm^−2^ on the same electrodes (Figure S13).

During the 500 mA cm^−2^ phase, the iR‐corrected cell voltage rises from ∼1.63 to ∼2.07 V (NF25) and ∼2.00 V (NF50), corresponding to raw voltage degradation rates of 6.57 mV h^−1^ (NF25) and 3.96 mV h^−1^ (NF50), Figure S13. These rates reflect ongoing structural reorganization of the catalyst layer alongside progressive vanadium dissolution (see below). Upon stepping to 1000 mA cm^−2^, both electrodes stabilize markedly: degradation rates fall to 0.76 mV h^−1^ (NF25) and 1.39 mV h^−1^ (NF50), indicating that the electrode reaches a structurally stable configuration after the initial vanadium‐loss phase. The iR‐corrected cell voltages plateau at ∼2.38 V (NF25) and ∼2.33 V (NF50) by end‐of‐test.

Post‐mortem ICP‐OES of the collected electrolytes confirms substantial vanadium dissolution, Table S4: 2673 µg L^−1^ (NF25) and 4898 µg L^−1^ (NF50), scaling approximately proportionally with the initial V_2_CT_x_ loading (NF50/NF25 ratio 1.83 vs. nominal 2.0), confirming that higher MXene content does not confer additional dissolution resistance. This dissolution is consistent with the potential‐dependent oxidative reconstruction of V_2_CT_x_ in alkaline aqueous environments recently characterized by Meng et al., who demonstrated that V_2_CO_2_ undergoes water nucleophilic attack at a low oxidation potential of −0.217 V vs. SHE via an OER‐like PCET pathway, confirming that the resulting vanadium oxide surface species are thermodynamically unstable at pH 14 [54].

Both composites also show elevated Ni and Fe leaching relative to pure NiFe (NF25: 2.1× Ni at 303 µg L^−1^, 1.2× Fe at 411 µg L^−1^; NF50: 2.8× Ni at 398 µg L^−1^, 1.8× Fe at 591 µg L^−1^; pure NiFe: 143 and 332 µg L^−1^), suggesting structural destabilization of the LDH layer upon vanadium loss.

Post‐mortem EDX of the recovered anodes, Figure S14, confirms near‐complete vanadium depletion: vanadium is at 0.10 ± 0.02 wt.% (at the EDX detection limit) in NF25 and 0.61 ± 0.02 wt.% in NF50 bulk. Post‐mortem XPS of the recovered anodes (Figure S15) detects no V2p signal in either NF25 or NF50, confirming complete surface vanadium depletion in both composites. The O1s signal remains present in both samples, confirming retention of the NiFe oxyhydroxide phase throughout the stability test. The high fluorine signal in both post‐mortem EDX spectra (30–38 wt.%) is consistent with ionomer distribution in the GDE catalyst layer. These results reframe the role of V_2_CT_x_: rather than acting as a permanent structural cocatalyst, V_2_CT_x_ functions as a sacrificial electronic modifier whose structural legacy, a modified LDH phase with altered Fe oxidation state distribution, disrupted morphology, and templated nucleation geometry, constitutes durable performance enhancement that persists throughout the 144 h test even after complete surface vanadium depletion.

## 4 Conclusions

This operando X‐ray absorption spectroscopy investigation reveals that V_2_CT_x_ actively participates in the oxygen evolution reaction through transient electronic coupling and sacrificial electronic modification, rather than serving as passive conductive support. This mechanistic understanding has important implications for catalyst design.

Our key findings establish that V_2_CT_x_ undergoes distinct transformations: (i) During hydrothermal synthesis at 120°C, V_2_CT_x_ oxidizes from low‐valent carbide (V^2+^/V^3+^) states, acting as a reducing agent that promotes ordered Fe_2_NiO_4_ formation while creating pre‐oxidized vanadium species. (ii) Under subsequent OER conditions, these species undergo further oxidation to establish a mixed‐valence V^3+^/V^4+^/V^5+^ system. Operando XANES reveals V K‐edge positions reaching 5483.1 eV (substantial V^5+^ character), while EXAFS demonstrates V─‐C bond transformation (2.079 Å) to V─O coordination (1.845 Å). Pre‐edge intensity ratios increase from 0.2 to 1.0, confirming VC_6_ → VO_6_ transformation.

The electronic coupling mechanism revealed by our operando studies provides new insights into catalyst design principles. The mixed‐valence V^3+^/V^4+^/V^5+^ system creates electronic pathways that suppress excessive nickel oxidation (Ni K‐edge shifts reduced from 2.8 to 2.3 eV) while maintaining favorable Fe^3+^ coordination for catalysis. Post‐mortem ICP‐OES, EDX, and XPS analysis confirms that vanadium undergoes progressive dissolution under extended alkaline operation, with complete surface depletion observed after 144 h. Yet the structurally modified NiFe electrodes retain their activity advantage throughout the full 144 h test, demonstrating that the synthesis‐stage electronic modification leaves lasting structural legacy: altered Fe oxidation state distribution, disrupted LDH morphology, and templated spinel nucleation, that sustains performance independently of vanadium presence. Improving vanadium retention under alkaline operating conditions, for example through protective surface passivation or selection of MXene compositions with greater alkaline corrosion resistance, represents a key direction for converting this transient coupling into a durable, permanent enhancement mechanism.

Electrocatalytic performance validates this mechanistic understanding: NiFe@V_2_CT_x_ composites achieve overpotentials of 304–309 mV at 10 mA cm^−2^ (93–98 mV improvement vs. NiFe) with 50% reduced charge transfer resistance. Optimal 25 wt.% V_2_CT_x_ loading balances electronic enhancement with active site accessibility. In zero‐gap AEM electrolyzers at 60°C, NF25 achieved a maximum cell efficiency of 83% with 80 mV lower cell voltage and 50% slower degradation rate (1.5 mV h^−1^) at 100 mA cm^−2^ than pure NiFe. Extended 144 h continuous testing at industrially relevant current densities, 72 h at 500 mA cm^−2^ followed by 72 h at 1000 mA cm^−2^, confirms that degradation rates decline sharply once the initial vanadium‐loss phase is complete, reaching as low as 0.76 mV h^−1^ (NF25) at 1000 mA cm^−2^, demonstrating that the sacrificial electronic modifier concept translates to durable device‐level performance.

This work provides the first operando spectroscopic characterization of the interplay between vanadium based MXene and carbonate intercalated NiFe‐LDH. The gradient heterostructure revealed by our analysis—consisting of regions with retained carbide character, mixed coordination zones, vanadium oxide surface species, and electronically coupled NiFeOOH active phase—demonstrates how controlled material evolution can create enhanced catalytic interfaces. This investigation challenges the prevailing assumption that MXene oxidation should be prevented in electrochemical applications. Instead, we demonstrate that controlled oxidation can create electronically active interfaces with superior catalytic performance. V‐O‐M bridges identified by EXAFS provide structural pathways for electron transfer during vanadium presence, establishing electronic communication that restructures the NiFe phase in a durable way.

The fundamental design principles established by this work, for instance electronic coupling through mixed‐valence redox interactions, synthesis stage structural templating, and the concept of sacrificial electronic modification, provide new directions for rational engineering of electrocatalysts. These mechanistic insights extend beyond the specific NiFe@V_2_CT_x_ system studied here, establishing general principles for designing composite catalysts where sacrificial component restructures the active phase during synthesis and early operation, leaving a lasting performance enhancement even after dissolution. This advanced understanding from passive support to active electronic mediator represents a promising approach in catalyst design with broad implications for electrochemical energy technologies.

## Author Contributions

**Merve Buldu‐aktürk**: formal analysis, investigation, writing – review and editing. **Can Kaplan**: investigation, methodology, formal analysis, writing – review and editing. **Karuppasamy Dharmaraj**: investigation, writing – review and editing, formal analysis. **Mailis Lounasvuori**: methodology, writing – review and editing, formal analysis. **Norbert Koch**: supervision, writing – review and editing, funding acquisition. **Bastian Schmiedecke**: formal analysis, writing – original draft, investigation, validation, data curation, writing – review and editing. **Axel Zuber**: investigation, writing – review and editing, formal analysis. **Johanna Rosen**: methodology, supervision, writing – review and editing, funding acquisition. **Thorsten Schultz**: investigation, methodology, formal analysis, writing – review and editing. **Ningjun Chen**: investigation, writing – review and editing. **Valeria Nicolosi**: writing – review and editing, methodology, supervision, funding acquisition. **Xuyun Guo**: methodology, formal analysis, writing – review and editing, investigation. **Michelle P. Browne**: conceptualization, funding acquisition, writing – review and editing, methodology, supervision, resources, project administration.

## Funding

We gratefully acknowledge the Helmholtz Association's Initiative and Networking Fund (Helmholtz Young Investigator Group (VH‐NG‐1719) for the funding. M.P.B greatly acknowledges support from the German Federal Ministry of Education and Research in the framework of the project Catlab (03EW0015A/B). M.P.B greatly acknowledges the support from the Daimler and Benz Stiftung under project number 32‐02/24. We acknowledge SOLEIL for provision of synchrotron radiation facilities, and we would like to thank A. Zitolo for assistance in using beamline “SAMBA” under proposal 20231879. The authors would like to address particular thanks to R. Schwiddessen, M. Tovar, K. Schwartzburg, and F. Ruske from the X‐Ray and Microscopy and Spectroscopy Corelabs of the Helmholtz Zentrum Berlin for providing access to their facility and training on the equipment. This work was supported by the European Union (ERC, MULTI2D, 101087713). V.N. and X.G. wish to thank the support of the Research Ireland‐funded AMBER Research Centre and the Research Ireland Frontiers for the Future award (Grant Nos. 12/RC/2278_P2 and 20/FFP‐A/8950, respectively). Meanwhile, the authors wish to thank SEAI (Award Number 24/RDD/01107) for their support. Furthermore, V.N. and X.G. wish to thank the Advanced Microscopy Laboratory (AML) in CRANN for the provision of their facilities and Clive Downing for assisting in the non‐routine maintenance of the microscopes. ICP‐OES measurements were carried out by the “Solar Fuels Testing Facility” laboratory of the Helmholtz Energy Materials Foundry (HEMF). We thank Iris Dobrandt for performing these measurements.

## Conflicts of Interest

The authors declare no conflict of interest.

## Supporting information




**Supporting File**: advs75676‐sup‐0001‐SuppMat.docx

## Data Availability

The data that support the findings of this study are available from the corresponding author upon reasonable request.
