# Supplementary material for: V2CTx MXene as a Sacrificial Promoter for NiFe Catalyst for Anion Exchange Membrane Electrolyzers
Source: Adv Sci (Weinh). 2026 May 18:e75676. Online ahead of print. doi: 10.1002/advs.75676 (PMC13336003; doi:10.1002/advs.75676)
Supplement: Supplementary file 1 — Supporting File: advs75676‐sup‐0001‐SuppMat.docx [file ADVS-9999-e75676-s001.docx]

**Supplementary Information**

**V₂CTₓ MXene as a Sacrificial Promoter for NiFe Catalyst for Anion Exchange Membrane Electrolyzers**


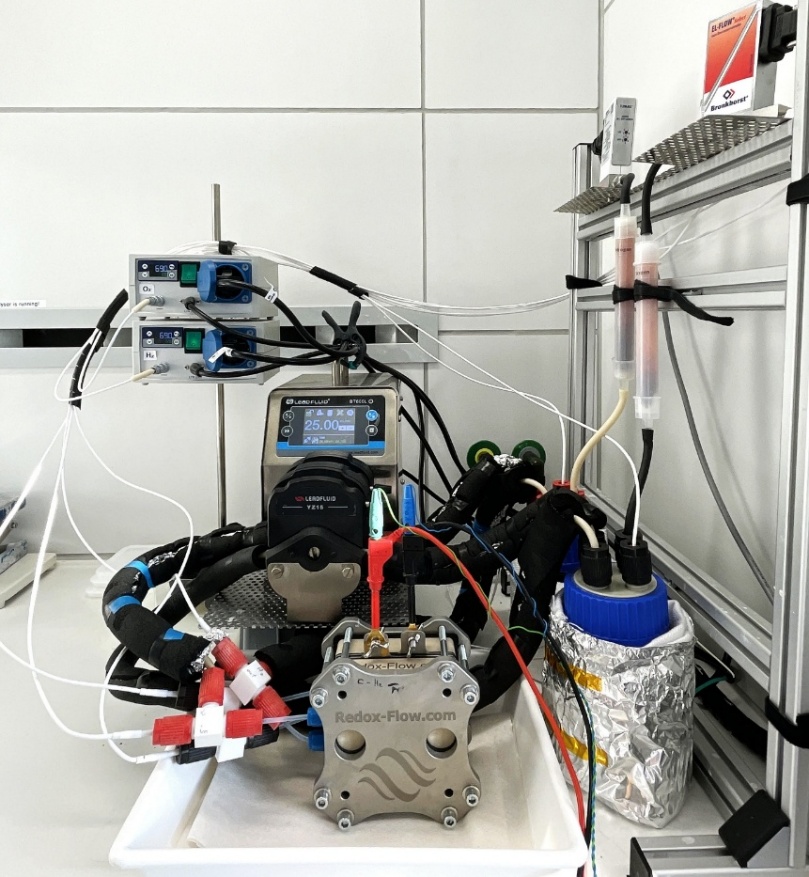


**Figure S1**. Picture of the AEM electrolyzer setup.


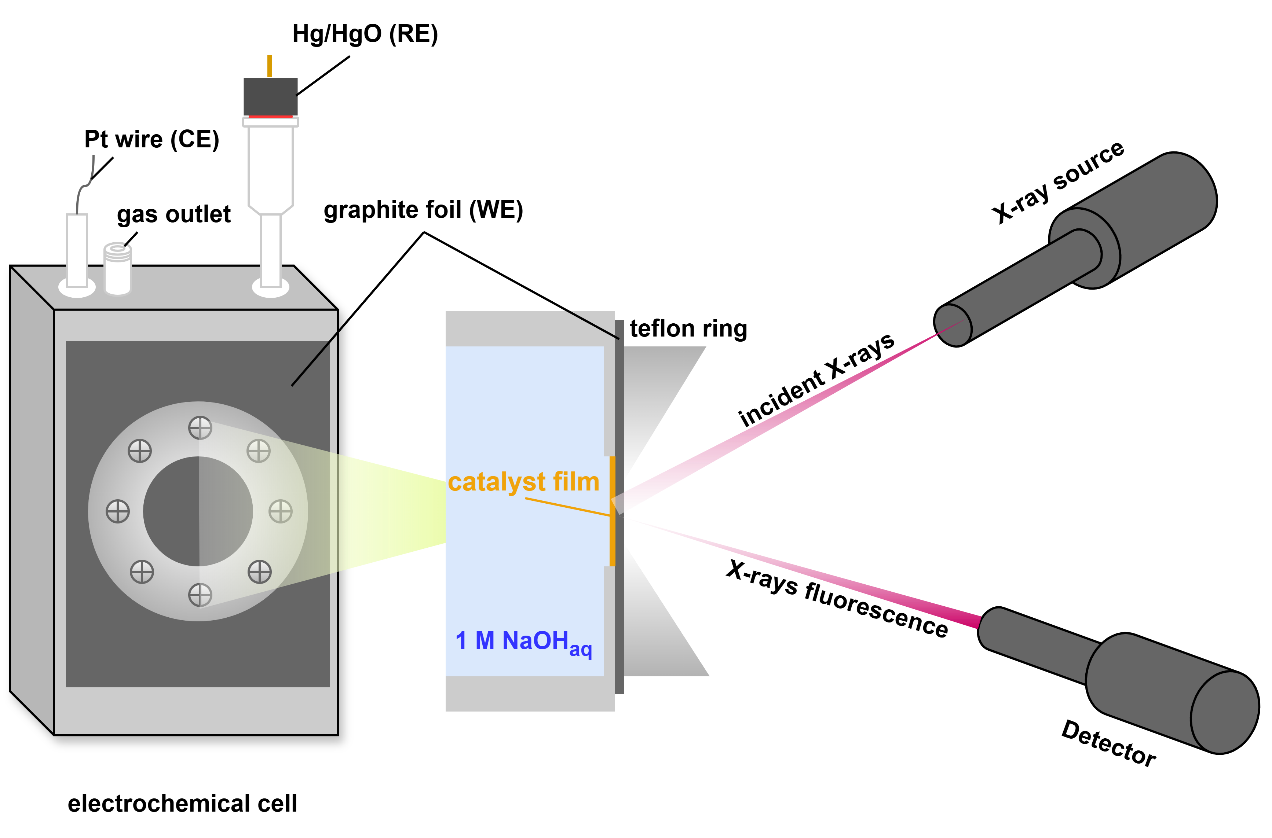


**Figure S2**. Schematic of the operando X-ray absorption spectroscopy experimental setup at SOLEIL beamline SAMBA.

**
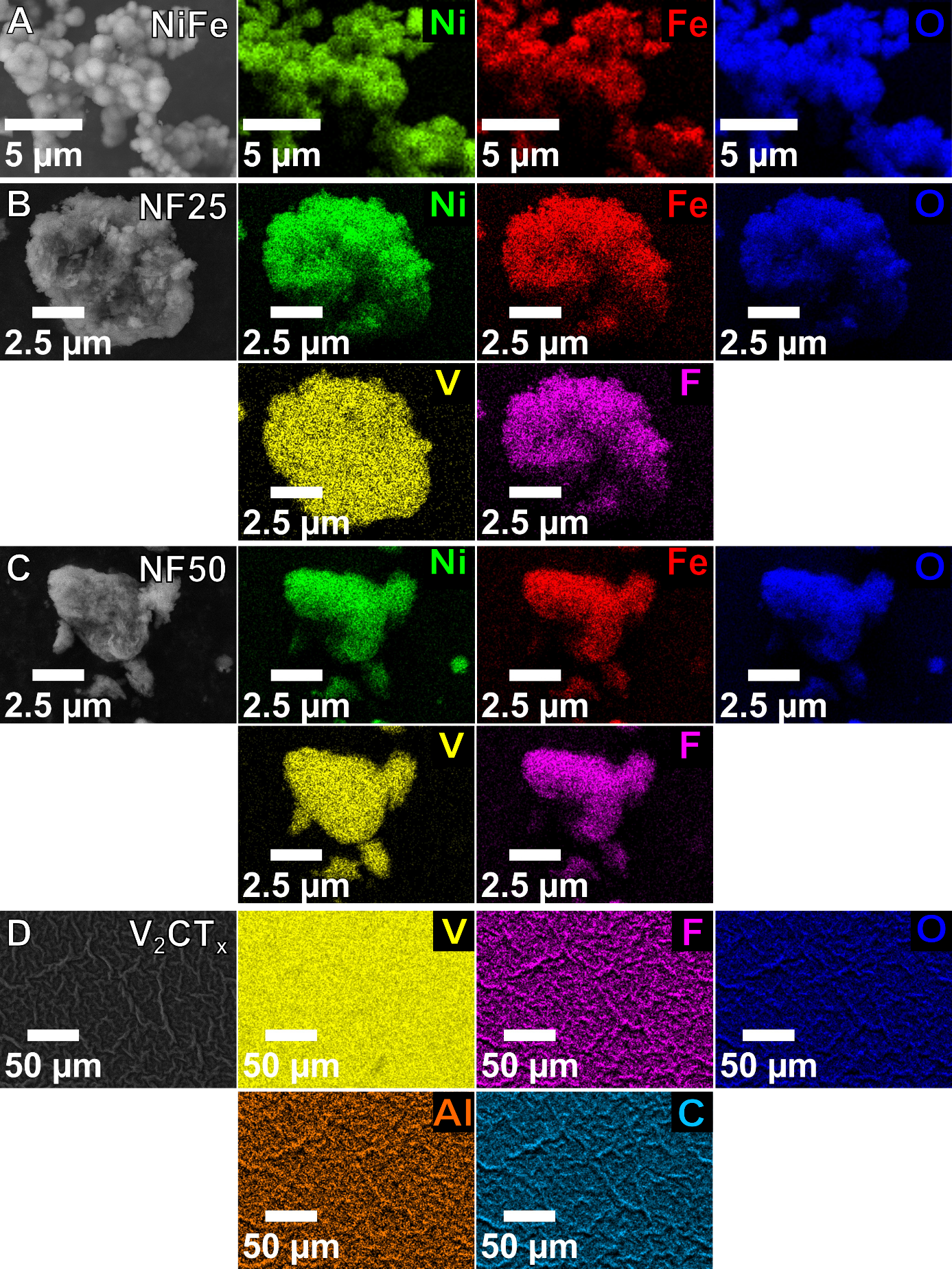
**

**Figure S3.** Energy-dispersive X-ray spectroscopy elemental maps for (A) NiFe, (B) NF25, (C) NF50, and (D) V_2_CT_x_ with vanadium (yellow), carbon (light blue), oxygen (dark blue), aluminum (orange), and fluorine (purple) distribution, confirming homogenous integration of V_2_CT_x_ within NiFe matrix in NF25 and NF50 (B, C).

**Table S1.** XRD peak assignments and observed d-spacings for pure NiFe-LDH, NF25/NF50 composites, and pure V_2_CT_x_ MXene. Peak positions were assigned from EVA background-subtracted diffractograms (Bruker Diffrac.EVA); d-spacings calculated by Bragg’s law (λ = 1.5406 Å, Cu Kα). Reference d-spacings for spinel NiFe_2_O_4_ taken from the reference structure (a = 8.339 Å). Notes in grey italics provide comparisons to external reference structures where relevant.

| **Sample** | **2θ obs (°)** | **d obs (Å)** | **(hkl)** | **Phase assignment & notes** |
| --- | --- | --- | --- | --- |
| **Pure NiFe-LDH** | | | | |
|  | 10.28 | 8.598 | *(003)* | CO_3_^2-^-NiFe-LDH (main phase) |
|  | 18.04 | 4.913 | *(006)* | SO_4_^2-^-LDH (minor phase), *implied d_003_ = 9.827 Å; not the (006) harmonic of the main CO_3_^2-^ phase* |
|  | ~30.4 | 2.934 | *unasgn.* | LDH higher-order harmonic or spinel (220), unresolved |
|  | 34.55 | 2.594 | *(012)* | CO_3_^2-^-NiFe-LDH |
|  | 40.46 | 2.228 | *(015)* | CO_3_^2-^-NiFe-LDH |
|  | 60.10 | 1.538 | *(110)* | CO_3_^2-^-NiFe-LDH |
| **NF25 / NF50 Composites** | | | | |
|  | 10.09 | 8.760 | *(003)* | CO_3_^2-^-NiFe-LDH (main phase), *d₀₀₃ = +0.16 Å vs pure NiFe-LDH; c-axis expansion* |
|  | 18.04 | 4.913 | *(006)* | SO_4_^2-^-LDH (minor phase) |
|  | 22.22 | 3.998 | *(008)* | V_2_CT_x_ (tentative), *theoretical d = 3.993 Å from (002); Δd = +0.005 Å* |
|  | ~30.4 | 2.934 | *unasgn.* | LDH higher-order harmonic or spinel (220), unresolved |
|  | 35.05 | 2.558 | *(012)* | CO_3_^2-^-NiFe-LDH |
|  | 38.50 | 2.336 | *(015)* | CO_3_^2-^-NiFe-LDH |
|  | 60.10 | 1.538 | *(110)* | CO_3_^2-^-NiFe-LDH |
|  | 63.20 | 1.470 | *(440)* | NiFe_2_O_4_ spinel (minor), *NiFe_2_O_4_ ref d = 1.474 Å; Δd = −0.004 Å; absent in pure NiFe-LDH* |
| **Pure V₂CTₓ MXene** | | | | |
|  | 5.53 | 15.968 | *(002)* | V_2_CT_x_ MXene, *d = 15.97 Å; c = 31.94 Å after delamination* |
|  | 9.10 | 9.710 | *(004)* | V₂CTₓ MXene |
|  | 11.70 | 7.558 | *(006)* | V₂CTₓ MXene |
|  | 23.50 | 3.783 | *(008)* | V₂CTₓ MXene |
| **Reference Spinel Reflections Absent in All Samples — Confirms Minor Spinel Content Only** | | | | |
| NF25/NF50 | - | - | *(311)* | NiFe_2_O_4_ spinel, absent, *expected 2θ = 35.68°, d_0_= 2.514 Å; not observed* |
| NF25/NF50 | - | - | *(400)* | NiFe_2_O_4_ spinel, absent, *expected 2θ = 43.37°, d_0_= 2.085 Å; not observed* |

**Derived lattice parameters:**

Pure NiFe-LDH: a = 3.077 Å, c = 25.794 Å, d₀₀₃ = 8.598 Å, interlayer gallery height = 3.83 Å. NF25/NF50 composites: a = 3.077 Å (unchanged), c = 26.279 Å, d_003_ = 8.760 Å, gallery height = 3.99 Å (Δd_003_ = +0.161 Å vs. pure NiFe-LDH).

SO_4_^-2^-LDH minor phase: implied d_003_ = 9.827 Å (from 18.04° (006) reflection).

The ∼30.4° shoulder is present in both pure NiFe-LDH and the composites and could not be unambiguously assigned; it is left unassigned in the main text.

**Figure S4. TEM characterization of pure V₂CTₓ and pure NiFe.** (A, B) HAADF-STEM images of V_2_CT_x_, showing the characteristic porous layered sheet morphology. (C, D) TEM images revealing the nanocrystalline V_2_CT_x_ sheet structure. (E) HRTEM image with lattice spacings indexed to V_2_CT_x_ (100) and (110) planes. (F) SAED pattern along the [001] zone axis confirming single-crystal quality and long-range stacking order of the delaminated V_2_CT_x_. (G–I) EELS elemental maps of pure V_2_CT_x_ (scale bar 20 nm): (G) ADF-STEM image, (H) vanadium, (I) carbon; co-localisation of V and C confirms intact carbide character. (J–K) HAADF-STEM images of pure NiFe showing the urchin-like nanorod aggregates. (L, M) TEM images of NiFe. (N-R) EELS elemental maps of pure NiFe (scale bar 20 nm): (L) ADF-STEM image, (M) carbon, (N) iron, (O) nickel, (P) oxygen; co-localisation of Fe, Ni, and O confirms the hydroxide/oxyhydroxide character of the pure NiFe phase in the absence of V_2_CT_x_.

**Figure S5. Additional TEM characterization of NF25.** (A) overview TEM image. (B, C) HAADF-STEM images showing NiFe clusters anchored on V_2_CT_x_ sheets. (D) HRTEM image showing nanocrystalline texture. (E) TEM image of the NF25 composite. (F-K) EELS elemental maps of a first NF25 interface region (scale bar 20 nm): (F) ADF-STEM image, (G) vanadium, (H) carbon, (I) iron, (J) nickel, (K) oxygen. (L-Q) EELS elemental maps of a second NF25 interface region confirming reproducibility of the elemental distribution: (L) ADF-STEM image, (M) vanadium, (N) carbon, (O) iron, (P) nickel, (Q) oxygen.

**Figure S6. Complete TEM characterization of NF50**. (A-C) HAADF-STEM showing the NF50 morphology; NiFe clusters are distributed across the V_2_CT_x_ sheet network analogously to NF25. (D) HRTEM image. (E, F) Overview TEM images. (G) HRTEM image with lattice spacings indexed to LDH (1 0 0) and ($\bar{1}$ 1 0) planes; no V_2_CT_x_ lattice fringes are observed. (H) SAED pattern of NF50 displaying diffuse polycrystalline rings indexed exclusively to LDH reflections (0 1 0), (0 1 3), and (0 1 5), confirming that V_2_CT_x_ long-range stacking order is equally disrupted at 50 wt.% loading as observed for NF25 (Figure 3H). (I–N) EELS elemental maps of a first NF50 interface region (scale bar 20 nm): (I) ADF-STEM image, (J) vanadium, (K) carbon, (L) iron, (M) nickel, (N) oxygen. (O-T) EELS elemental maps of a second NF50 interface region confirming reproducibility: (O) ADF-STEM image, (P) vanadium, (Q) carbon, (R) iron, (S) nickel, (T) oxygen. In both regions, V and C are confined to the MXene sheet domains while Fe, Ni, and O are sharply co-localized in the adjacent LDH phase, mirroring the interfacial architecture observed in NF25 and confirming that the V_2_CT_x_-NiFe contact geometry is preserved across both composite loadings.

**Figure S7.** (A) O1s and V2p core level raw spectra, and (B) Ni Auger core level spectra of NiFe, NF25, and NF50.

**Figure S8.** Chronopotentiometric stability curves at 10 mA cm^-2^ including V_2_CT_x_ MXene and Ni felt controls. The rapid initial potential decrease observed for V_2_CT_x_ is attributed to surface reconstruction of the MXene under anodic conditions, consistent with the oxidation of low-valent vanadium carbide surface terminations (V^n+^, n < 4) to higher oxidation states, as supported by the XPS evidence for mixed vanadium oxidation states discussed in 3.1.2. This surface reconstruction is expected to be substantially suppressed in the NF25 and NF50 composites, where V_2_CT_x_ nanosheets are encapsulated by the hydrothermally grown NiFe-LDH phase, consistent with the XRD observation of disrupted MXene stacking order in both composite samples (Paragraph 3.1.2). V_2_CT_x_ and Ni felt are shown here as reference baselines; the focused performance comparison among NiFe-LDH, NF25, and NF50 is presented in Figure 5E of the main text.

**A**

**B**

**C**

**D**

**Figure S9.** Nyquist plots of NiFe, NF25, NF50 and V_2_CT_x_, measured at applied potentials of 1.5, 1.55, 1.6 and 1.65 V vs. RHE.

**A)**

**B)**

**D)**

**d)**

**C)**

**E)**

**F)**

**Figure S10. Electrochemically active surface area determination.** (A-D) Exemplary cyclic voltammograms (CVs) at scan rates 10-100 mV s^-1^ in the non-faradaic region for (A) pure NiFe, (B) NF25, (C) NF50 and (D) pure V_2_CT_x_. (E) Linear relationship between current density and scan rate, with slopes proportional to double-layer capacitance *C_dl_*. (F) Electrochemically active surface areas calculated from *C_dl_* values using a specific capacitance of 0.04 mF cm^-2^ for transition metals in alkaline electrolyte.

**Figure S11. Operando X-ray absorption spectroscopy**. XANES spectra showing the (A) Ni, (B) Fe K-edge and pre-edge of NiFe, (C) V K-edge and pre-edge of V_2_CT_x_, and the (D) Ni, (E) Fe, and (F) V K-edge and pre-edge of NF50 under various applied OER overpotentials.

**Table S2.** First shell fit results Values highlighted in red are excluded from physical interpretation. For V_2_CT_x_ ‘after OER’: the V K-edge signal was too attenuated following vanadium dissolution to yield a physically meaningful fit (S_0_^2^ = -9.023, unphysical). For NF50 V at 0.6 V and V ‘after OER’: structural heterogeneity arising from mixed V-C/V-O coordination at high vanadium loading produces a featureless EXAFS signal that cannot be modelled with a single coordination shell (S_0_^2^ > 9, unphysical). These conditions are consistent with the incomplete oxidation and phase heterogeneity discussed in Paragraph 3.3.1 and 3.3.2. All remaining entries satisfy standard quality criteria (R-factor < 0.02, positive σ^2^, ΔE_0_ within ±15 eV). Physical interpretation throughout the manuscript is restricted exclusively to bond distances R(M-O) from the non-flagged entries.

| **Material** | **Edge - potential** | **S_0_²** | **σ²** | **E_0_** | **ΔR** | **R-factor** | **R [M-O]** |
| --- | --- | --- | --- | --- | --- | --- | --- |
| NF25 | Fe - exsitu  Fe - 0.6 V  Fe - after  Ni - exsitu  Ni - 0.6 V  Ni - after | 3.288  2.735  1.390  3.569  3.683  4.349 | 0.00349  0.00344  0.00333  0.00103  0.00455  0.00324 | 2.253  -4.561  -0.036  -1.833  -3.755  -3.743 | -0.6474  -0.16412  -0.07527  -0.03753  -0.19490  -0.04957 | 0.019  0.012  0.018  0.007  0.015  0.008 | 2.03526  1.93588  2.02473  2.06247  1.90510  2.05043 |
| V_2_CT_x_ | V - exsitu  V - 0.45V  V - after | 3.532  1.773  -9.023 | 0.00244  0.00962  0.00385 | -5.649  14.285  -16.824 | -0.07291  -0.00696  -0.25843 | 0.018  0.019  0.019 | 2.02709  2.09304  1.84157 |
| NF50 | V - exsitu  V - 0.6V  V - after | 0.890  9.64919  11.067 | 0.00070  0.00522  0.00705 | -1.037  -24.743  -23.635 | -0.05150  -0.02050  -0.01612 | 0.020  0.015  0.015 | 2.04850  2.07950  2.08388 |

**Figure S12.** Ex-situ EXAFS spectra of the as-prepared material films.

Table S3. Energy consumption, electrolysis power and cell efficiency of NiFe, NF25, and NF50 with and without *iR* correction at 100 mA cm^–2^ in 1.0 M NaOH at 60 °C.

| **Catalyst** | **Energy consumption**  **[kWh Kg^–1^]** | **Electrolyzer power**  **[W cm^–2^]** | **Cell efficiency**  **[%]** |
| --- | --- | --- | --- |
| NiFe (0 % iR correction) | 47 | 0.162 | 77 |
| NiFe (100 % iR correction) | 47 | 0.16 | 78 |
| NF25 (0 % iR correction) | 45 | 0.16 | 82 |
| NF25 (100 % iR correction) | 44 | 0.152 | 83 |
| NF50 (0 % iR correction) | 45 | 0.155 | 81 |
| NF50 (100 % iR correction) | 45 | 0.153 | 82 |

**Figure S13.** iR-corrected cell voltage vs. time for NF25 and NF50 anodes in zero-gap AEM electrolyzer during 144 h continuous operation: 72 h at 500 mA cm^-2^ (left region) followed by 72 h at 1000 mA cm^-2^ (right region) on the same electrodes. High-frequency resistance (HFR) measured by GEIS was used for ohmic correction throughout. Operating conditions: 1.0 M NaOH, 60 °C, PiperION A80 AEM, 1.0 mg cm^-2^ anode loading.

**Table S4.** ICP-OES analysis of electrolytes collected after 144 h AEM electrolyzer operation.

| **Sample** | **Ni [µg L^-1^]** | **Fe [µg L^-1^]** | **V [µg L^-1^]** |
| --- | --- | --- | --- |
| NiFe | 143 | 332 | 0 |
| NF25 | 303 | 411 | 2673 |
| NF50 | 398 | 591 | 4898 |

**Figure S14.** Post-mortem Energy Dispersive X-ray spectroscopy of recovered NF25 and NF50 anodes after 144 h stability test. (A) NF25, shown with Ni (green), Fe (orange), V (purple), and O (blue), with corresponding Table showing elemental distribution in weight %. (B) NF50 shown with Ni (green), Fe (orange), V (purple), and O (blue), with corresponding Table showing elemental distribution in weight %. Elemental quantification by EDX is semi-quantitative only; values are reported for comparative purposes. The high F signal (30-38 wt%) reflects ionomer distribution in the GDE catalyst layer and is excluded from the metal/oxygen analysis.

**Figure S15.** Post-mortem X-ray photoelectron spectroscopy of recovered NF25 and NF50 anodes after 144 h of continuous AEM electrolyzer operation. O1s and V2p core level spectra are shown for NF25 and NF50. In both composites, the V2p signal is completely absent, confirming total surface vanadium depletion after extended alkaline operation. The O1s signal remains present in both samples, consistent with retention of the NiFe oxyhydroxide phase.
